# Comparative proteomic study of Arabidopsis mutants *mpk4* and *mpk6*

**DOI:** 10.1038/srep28306

**Published:** 2016-06-21

**Authors:** Tomáš Takáč, Pavol Vadovič, Tibor Pechan, Ivan Luptovčiak, Olga Šamajová, Jozef Šamaj

**Affiliations:** 1 Centre of the Region Haná for Biotechnological and Agricultural Research, Faculty of Science, Palacký University, Šlechtitelů 27, 783 71 Olomouc, Czech Republic; 2 Institute for Genomics, Biocomputing & Biotechnology, Mississippi Agricultural and Forestry Experiment Station, Mississippi State University, MS 39759, USA

## Abstract

Arabidopsis MPK4 and MPK6 are implicated in different signalling pathways responding to diverse external stimuli. This was recently correlated with transcriptomic profiles of Arabidopsis *mpk4* and *mpk6* mutants, and thus it should be reflected also on the level of constitutive proteomes. Therefore, we performed a shot gun comparative proteomic analysis of Arabidopsis *mpk4* and *mpk6* mutant roots. We have used bioinformatic tools and propose several new proteins as putative MPK4 and MPK6 phosphorylation targets. Among these proteins in the *mpk6* mutant were important modulators of development such as CDC48A and phospholipase D alpha 1. In the case of the *mpk4* mutant transcriptional reprogramming might be mediated by phosphorylation and change in the abundance of mRNA decapping complex VCS. Further comparison of *mpk4* and *mpk6* root differential proteomes showed differences in the composition and regulation of defense related proteins. The *mpk4* mutant showed altered abundances of antioxidant proteins. The examination of catalase activity in response to oxidative stress revealed that this enzyme might be preferentially regulated by MPK4. Finally, we proposed developmentally important proteins as either directly or indirectly regulated by MPK4 and MPK6. These proteins contribute to known phenotypic defects in the *mpk4* and *mpk6* mutants.

MAPKs are important signalling molecules which are involved in transduction of signal derived from many environmental and developmental stimuli^1^. MAPK signalling results in gene activation triggering appropriate defense responses or in the activation or repression of developmentally-regulated proteins modulating plant growth and development^2^. The complete sequencing and annotation of the Arabidopsis genome resulted in the identification of 20 MAPK-encoding genes, while *MPK4*, *MPK6* and *MPK3* are the most studied ones and they play dominant roles in the transduction of stress and developmental signal as well as in the cross-talk with other signaling pathways^2^.

MPK4 and MPK6 are commonly activated by cold and salt stress downstream of MEKK1 (MAP3K) and MKK2 (MAP2K) in Arabidopsis^3^, while MPK4 via this pathway also negatively regulates salicylic acid (SA) and ROS production^4^. MEKK1-mediated activation of MPK6 (together with MPK3) was reported also via MKK4/MKK5 in the response to bacterial elicitor flagellin. This signaling cascade affects expression of defense related genes^5^. The MKK4-MPK6 module also phosphorylates and activates ACC synthases ACS2 and ACS6 which are key enzymes for ethylene synthesis^6^. Additionally, MPK6 is specifically activated by MKK3 in response to jasmonic acid (JA) and negatively regulates the JA signalling pathway^7^. MPK6 is also activated by the pathway that includes ANP1 (MAP3K)–MKK4/MKK5 upon hydrogen peroxide treatment^8^. In contrary, MPK4 is activated by MEKK1 (MAP3K)–MKK2 (MAP2K) pathway under oxidative stress^9^.

Along with roles of MPK4 and MPK6 in various stress responses, they are involved in multiple developmental processes. Thus, MPK6 is involved in embryogenesis^10^, stomata formation^11^, cell division plane orientation and root development^12^ and it is localized in the secretory *Trans*-Golgi network (TGN) vesicles and plasma membrane. MPK4 controls cell division, root growth and formation of root hairs^13^,^14^.

An absence of particular members of the MAPK cascade in Arabidopsis single and double knockout mutants can lead to considerable molecular, physiological, developmental and phenotypic changes, and it can modify their stress responses^3^,^10^. However, there is only very scarce information about the proteome wide effects resulting from genetically-affected MAPK signaling in plants^15^.

Roots of *mpk4* mutant have altered organization of cortical microtubules leading to radial root expansion and adverse effects on root hair morphogenesis^13^. At the subcellular level, the *mpk4* mutant showed aberrant spindle and phragmoplast formation and drastically delayed or abortive mitosis and cytokinesis^14^. Additionally, hydrogen peroxide accumulation, reduced expression of auxin inducible marker genes and elevated levels of mRNA encoding stress response proteins was observed in the seedlings of *mpk4* mutant^4^. Detailed analyses of *mpk6* knock-out mutants showed root phenotypes, which are consequences of ectopic cell divisions and aberrant orientation of cell division, resulting in disordered root cell files in the *mpk6* mutants^12^. Here, we have studied major changes associated with MPK4 and MPK6 deficiency in Arabidopsis roots on the proteome level. We aimed to find protein candidates contributing to specific root phenotypic features of *mpk4* and *mpk6* mutants. In addition, we also focused on proteome changes associated with oxidative stress and defense responses in these two mutants.

## Results

### Overview of root proteomes of Arabidopsis mpk4 and mpk6 mutants

**Figure 1:**
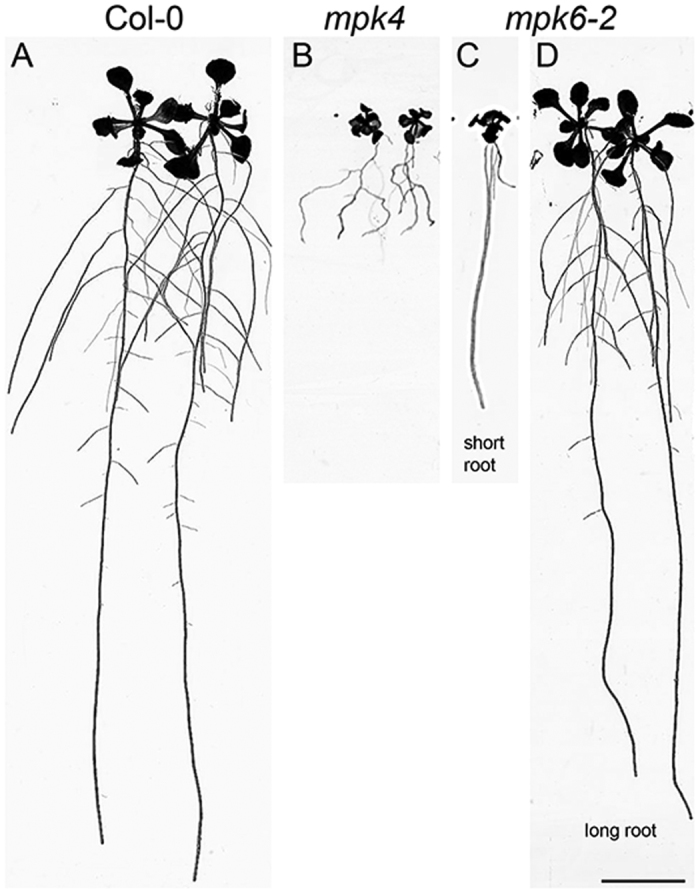
Representative pictures of 14 days old wild type plants Col-0 (**A**), *mpk4* (**B**) and *mpk6-2* short (**C**) and long root (**D**) mutants used for proteomic and biochemical analyses. Bar: 1 cm.

We performed proteomic analyses of *mpk4* and *mpk6* mutant roots in direct comparison to the Col-0 wild type. Representative pictures of analyzed mutants are provided in Fig. 1. This shot gun MS/MS-based proteomic analysis identified in average 437 proteins in the *mpk4* and 444 proteins in the *mpk6* mutant. All proteins with statistically significant changes in their abundances between wild type and mutant plants were selected using one-way Anova test. They are provided in Supplementary Tables S1 and S2.

First, we compared the numbers of differentially regulated proteins in both mutants (Supplementary Table S3). Only proteins with change higher than 1.5 fold were considered in this study. We detected 63 differentially abundant proteins in the *mpk4* mutant when compared to Col-0 wild type seedlings, while there were 32 differentially abundant proteins in the *mpk6* mutant. In addition, several proteins (33 and 19 for *mpk4* and *mpk6,* respectively) were detected only in one sample, either in the wild type, or in the one of the mutants, indicating that in such cases their abundance was below the detection sensitivity limit. Altogether, we have found 96 and 51 differentially abundant proteins in the roots of *mpk4* and *mpk6* mutants, respectively. These data might indicate that MPK4 deficiency has broader influence on Arabidopsis basal root proteome as compared to MPK6.

**Figure 2:**
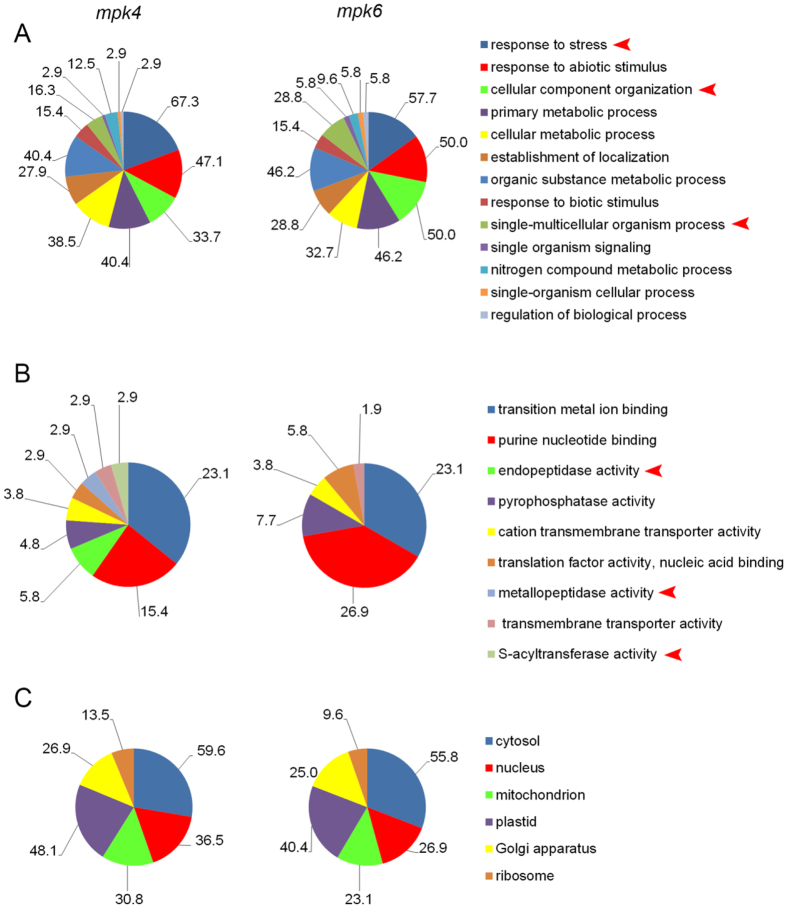
Comparison of gene ontology classification (at 3^rd^ level) of *mpk4* and *mpk6* mutant root differential proteomes according to (**A**) biological process, (**B**) molecular function, (**C**) cellular compartment. Arrowheads indicate most prominent differences between the mutants.

In order to functionally classify these proteins in differential proteomes of both mutants, we performed analyses of known and predicted functional protein association networks using STRING^16^ (Supplementary Figs S1 and S2) and gene ontology (GO) annotation according to biological process, molecular function and cellular compartment using Blast2Go^17^ software (Fig. 2A–C). These analyses revealed that *mpk4* and *mpk6* mutants did not substantially differ in the number of GO annotations detected in differential proteomes according to biological function at the 3^rd^ level of ontology (Fig. 2A). Nevertheless, some interesting differences, including different number of proteins in the two mutants, have been found in GO annotations in terms of response to stress, cellular component organization and single multicellular organism process (Fig. 2A) as well as in terms of molecular functions (Fig. 2B). On the other hand, no changes were detected in GO annotations in terms of subcellular localization (Fig. 2C).

We also performed a GO annotation analysis on whole proteomes of *mpk4* and *mpk6* roots (Supplementary Figs S3–S5). In this case, only minor differences were observed between whole proteomes of the mutants.

Next, we searched for proteins which abundances were commonly changed in both mutants compared to wild type (Supplementary Table S4). Eight differentially abundant proteins were detected in both *mpk4* and *mpk6* roots. Two of them showed decreased abundance in both mutants (arabinogalactan protein 31 and profilin 2) and three other proteins had increased abundance in both mutants (glyceraldehyde 3-phosphate dehydrogenase, TRAF-like family protein and translational initiation factor 4A-1). These small numbers of commonly regulated proteins show that root proteomes of the mutants are quite different.

### Does MPK3 compensate for missing MPK6 in roots of the mpk6 mutant?

**Figure 3:**
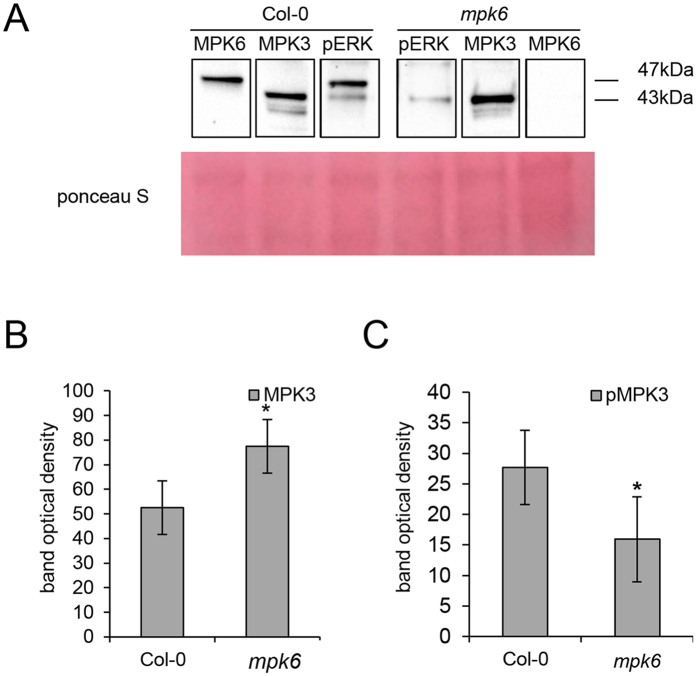
Imunoblotting analysis of MPK3 activity in the *mpk6* mutant using phospho-specific pERK antibody. (**A**) Representative immunoblots of wild type and *mpk6* mutant roots probed with anti-MPK3 (lane MPK3), anti-MPK6 (lane MPK6) and anti-phospho-p44/42 (pERK) antibodies. (**B**) Quantification of the band optical density corresponding to MPK3 in (**A**). (**C**) Quantification of the band optical density corresponding to phosphorylated MPK3 (pMPK3) (band of 43 kDa in lane pERK in **A**) in wild type and *mpk6* mutant roots. Error bars are standard deviations calculated from 3 biological replicates. Stars indicate significant difference at p ≤ 0.05 according to Student *t*-test.

MPK3 and MPK6 show high level of functional redundancy in response to external stimuli^8^ and in regulating plant development^18^. It was also found that MPK3 activities might be increased in *mpk6* mutants in response to various external stimuli. In this study, we evaluated the MPK3 abundance and activity in the roots of the *mpk6* untreated mutant using affinity purified antibodies against MPK3 and phosphorylated mammalian ERK1/2 (phospho-p44/42, pERK) recognizing pTEpY-dual phosphorylation motif (Fig. 3), preferentially in MPK6 and MPK3. We detected increased abundance of MPK3 in the *mpk6* mutant as compared to the Col-0 wild type (Fig. 3A,B). By using pERK antibody we detected two bands in Col-0 wild type roots, corresponding to phosphorylated MPK6 and MPK3, respectively. As expected, *mpk6* mutant showed only a single band, corresponding to the activated MPK3 which was, however, less prominent in the mutant as compared to the Col-0 wild type (Fig. 3A,C). These data suggest that differences in the root proteome composition of the *mpk6* mutant might be, at least partially, attributed also to the higher abundance but lower activity of MPK3 in the roots of this mutant.

### Prediction of putative MPK4 and MPK6 targets

Proteomic analyses performed on the loss-of-function and knockout mutants can help to identify both direct and indirect targets of MAPKs. The interaction of MAPKs with their direct protein targets is conditioned by the presence of MAPK docking site, MAPK-specific phosphorylation site and common subcellular localization with MAPKs. Therefore, we analyzed proteomically-identified proteins of *mpk4* and *mpk6* mutant roots for such motifs and gained information about localization of these putative target proteins. Proteins containing both docking and phosphorylation motifs are listed in Tables 1 and 2. Details of prediction including probabilities, scores, putative phosphorylation sites and amino acid sequences of the docking domains are provided in Supplementary Tables S5–S8. We have found 17 and 8 putative target proteins in the *mpk4* and *mpk6* mutants, respectively. The localization of some putative target proteins corresponded with the published localization of MPK4 and MPK6 in Arabidopsis^12^,^14^,^19^. These were for example elongation factor EF-2, uncharacterized protein (gi:240254562) and mRNA decapping complex VCS in the *mpk4* mutant as well as CDC48A and phospholipase D alpha 1 in the *mpk6* mutant, while heat shock protein 70–3 was found in both mutants.

### Abundance of abiotic stress related proteins in mpk4 and mpk6 mutants

**Figure 4:**
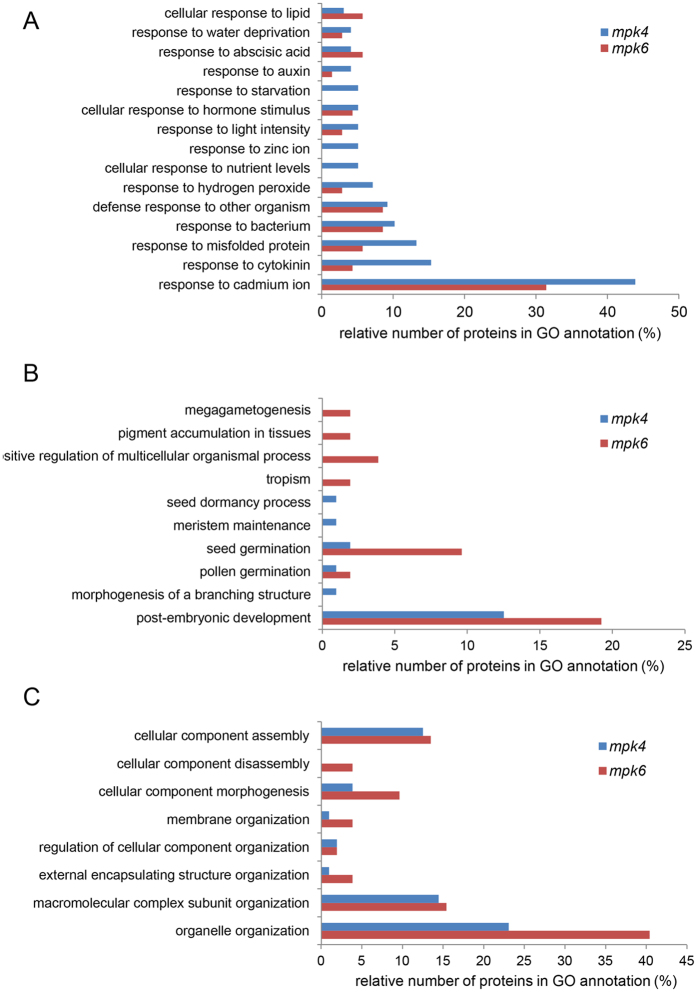
Comparison of gene ontology categories related to (**A**) response to external stimuli (6^th^ level of ontology), (**B**) single-multicellular organism process (5^th^ level of ontology) and (**C**) cellular component organization (4^th^ level of ontology) between *mpk4* and *mpk6* mutants.

Considerable differences were found between the mutants in proteins annotated by GO “response to stress” (Fig. 2A). Therefore we focused in detail on GO annotations related to response to external stimuli in the differential proteomes of the mutants (Fig. 4A). Some GO annotation categories such as response to starvation, zinc ion and nutrient levels were not detected in the *mpk6* mutant but they were present in the *mpk4* mutant. Further, significantly more differentially-regulated proteins in the *mpk4* mutant, as compared to the *mpk6* mutant, were annotated for response to cadmium, cytokinin, misfolded protein, auxin and hydrogen peroxide. These analyses suggested that related physiological responses might be more altered in the *mpk4* mutant in comparison to the *mpk6* mutant.

Both MPK4 and MPK6 are important signalling proteins during oxidative stress, albeit being activated by different signalling modules^8^,^9^. Roots of the *mpk4* mutant showed decreased abundance of 7 proteins (monodehydroascorbate reductase, L-ascorbate peroxidase 1, L-ascorbate peroxidase S, peroxidase family protein, glutathione S-transferase phi 8, nucleoside diphosphate kinase 1 and peroxidase 27) involved in oxidative stress response and antioxidant defense as compared to the Col-0, while other 4 proteins (glutathione S-transferase F2, glutathione S-transferase TAU 19, monodehydroascorbate reductase (NADH) and catalase 3) were upregulated. Consistently, bioinformatic analysis of interaction networks of proteins with increased abundance in the *mpk4* mutant showed the downregulation of cluster composed of enzymes involved in ascorbate-glutathione cycle (Supplemental Fig. 1A). Surprisingly, in the *mpk6* mutant, we detected only one antioxidant enzyme, monodehydroascorbate reductase (NADH), which is downregulated in comparison to the wild type control. These data indicate that the Arabidopsis antioxidant defense might be regulated predominantly by MPK4.

**Figure 5:**
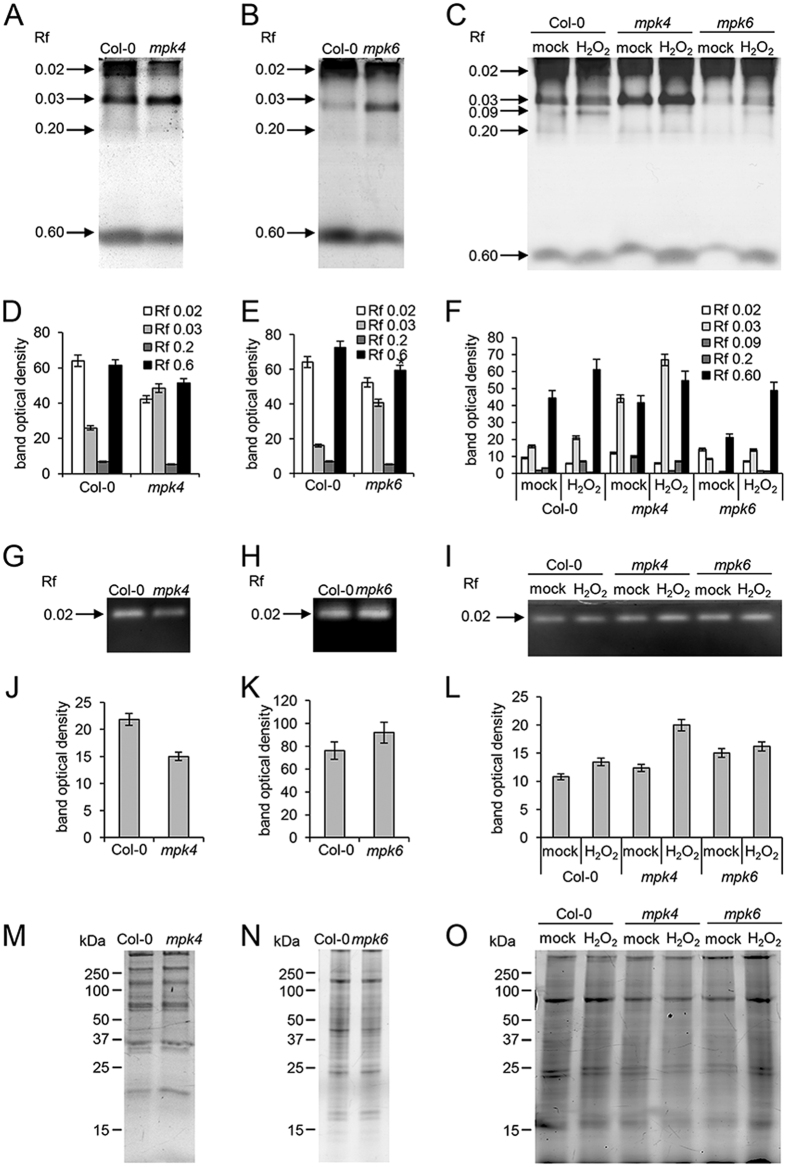
Analyses of peroxidase and catalase enzymatic activities in the *mpk4*, *mpk6* mutant and in the wild type. (**A–C**) Peroxidase specific activity staining in *mpk4* and *mpk6* mutant roots without any treatment (**A,B**) or after 1 day long incubation in liquid 1/2 MS medium with or without 150 μM hydrogen peroxide (**C**). (**D–F**) Quantification of the bands optical densities in **A**, **B** and **C**. (**G–I**) Catalase specific activity staining in *mpk4* and *mpk6* mutant roots without any treatment (**G,H**) or after 1 day long incubation in liquid 1/2 MS medium without or with 150 μM hydrogen peroxide (**I**). (**J–L**) Quantification of the bands optical densities in **G, H** and **I**. (**M–O**) Respective loading control represented by protein visualization using stain free technology (Biorad) on SDS PAGE gels. Error bars are standard deviations calculated from 3 biological replicates.

In order to monitor the activities of hydrogen peroxide decomposing enzymes in *mpk4* and *mpk6* roots, we carried out specific activity staining of peroxidases and catalase on native PAGE gels. We detected four isozymes of peroxidases in both mutants and wild type. Three isozymes (with Rf 0.02, 0.03 and 0.6) showed substantial differences between Col-0 and the mutants (Fig. 5A,B,D,E). The changes in peroxidase isozymes were similar in both mutants when compared to the wild type. To analyze activities of peroxidase isozymes under oxidative stress, we exposed the wild type, *mpk4* and *mpk6* seedlings to 150 μM hydrogen peroxide (in liquid 1/2 MS for 24 h). The total peroxidase activity was apparently higher in the *mpk4* mutant as compared to the wild type after incubation in mock treatment (liquid 1/2 MS medium), while it was reduced in the *mpk6* mutant (Fig. 5C,F). Hydrogen peroxide activated one peroxidase isozyme with Rf 0.09 in Col-0 control while the other isozymes remained unaffected. On the other hand, the overall peroxidase activity was increased in response to hydrogen peroxide in both mutants (especially isozymes with Rf 0.02 and 0.03 in Fig. 5C,F).

Further, we observed significantly lower total catalase activity in the *mpk4* mutant while it was slightly elevated in the *mpk6* mutant when compared to wild type (Fig. 5G,H,J,K). On the other hand, catalase activity significantly increased in response to the hydrogen peroxide treatment only in the *mpk4* mutant. These results show that *mpk4* and *mpk6* mutant roots have similar basal and hydrogen peroxide-induced peroxidase isozyme pattern compared to Col-0. In contrast to peroxidase, total catalase activity in control conditions and also in response to hydrogen peroxide showed noticeable differences between the mutants.

### mpk4 and mpk6 mutants show differences in defense related proteins

MPK4 and MPK6 are activated by different MAPK cascades in response to the pathogen attack^20^. Therefore, it is challenging to decipher whether this difference affects the composition of the defense related proteins in the *mpk4* and *mpk6* mutants.

We detected a canonical defense inducible protein, root specific beta-1,3-endoglucanase^21^ and DNA topoisomerase (named as NAI2)^22^ as upregulated in the *mpk4* mutant roots, supporting well-known activation of pathogen defense in the *mpk4* mutant^23^. These proteins, together with JA responsive 1, form an interaction cluster showing increased abundance in the *mpk4* mutant as revealed by STRING functional protein association network analysis (Supplementary Fig. S1A). Similarly, some other pathogen inducible proteins were upregulated as well. These included mitochondrial voltage-dependent anion channel^24^, short-chain dehydrogenase reductase 3^25^ and finally Kunitz trypsin inhibitor, which acts as antagonist of cell death triggered by phytopathogens^26^. Strikingly, not all pathogenesis related proteins showed constitutively increased abundances in the *mpk4* mutant. Thus, glutathione S-transferase 8 known as marker for early defense response^27^ and heat stable protein 1 with antimicrobial activity^28^ were downregulated (Supplementary Table S1). Another two downregulated proteins in the *mpk4* mutant are involved in virus multiplication. The TOMV RNA binding protein binds to tomato mosaic virus genomic RNA and inhibits its multiplication^29^. HSP70–3 interacts with Turnip mosaic virus RNA-dependent RNA polymerase (RdRp) and is a component of a replicase complex regulating RdRp function^30^. HSP70–3 was also shown to regulate plant immune responses against bacteria in interaction with co-chaperone SGT1^31^. Therefore, MPK4 might modulate virus multiplication.

In addition to these proteins, jasmonic acid (JA) responsive protein 1 (JR1) was less abundant in the *mpk4* roots (Supplementary Table S1). Since JR1 is accumulating during JA response^32^ it indicates downregulation of JA signalling pathway in the *mpk4* mutant. On the other hand, JA-inducible lipoxygenase1 (LOX1), which is also involved in JA biosynthesis^33^ was detected uniquely in the *mpk6* mutant (Supplementary Table S2), suggesting upregulation of JA signalling in this mutant. Altogether, these data indicate different regulation of JA signalling by MPK6 and MPK4.

The *mpk6* mutant showed also increased abundances of NADP-dependent malic enzyme 2, an enzyme important for pathogen defense^34^ and CDC48 protein responding to pathogen infection and controlling movement of Tobacco mosaic virus^35^. Finally, the HSP70–3, unlike in the *mpk4*, was upregulated in the *mpk6* mutant (Supplementary Table S2).

### Differential abundance of scaffold proteins RACK1 and AtRem1.3 in mpk6 and mpk4 mutants

WD-40 repeat ArcA-like protein (RACK1) was detected uniquely in the *mpk*6 mutant (Supplementary Table S2), while it was under the detection threshold in the *mpk4* mutant. RACK1 functions as a scaffold protein linking MEKK1-MKK4/MKK5-MPK3/MKP6 cascade during immune pathway activated by pathogen-secreted proteases^36^. In addition, RACK1 has a multiple roles in plant development and hormone responses^37^. According to our results, MPK6 may negatively regulate the expression of RACK1 in Arabidopsis.

Another scaffold protein involved in plant-microbe interactions is remorin family protein (AtRem1.3) which showed decreased abundance in the *mpk4* mutant (Supplementary Table S1). AtREM1.3 is differentially phosphorylated upon treatment with bacterial elicitors and likely plays a role as scaffold protein in plant innate immunity^38^. Our data provide a first evidence of possible co-regulation of AtRem1.3 and MAPK signalling.

### Plant developmental proteins in mpk4 and mpk6 mutants

Considerable differences between *mpk4* and *mpk6* mutants have been found in GO category named single-multicellular organism process (Fig. 2A). Further, we focused on downstream GO annotations within the GO hierarchy (Fig. 4B). This GO annotation contains proteins implicated mainly in developmental processes. According to this evaluation, root proteome of *mpk6* contained proteins involved in megagametogenesis, pigment accumulation in tissues and tropism, while such GO annotations were not present in the root proteome of *mpk4* mutant. In addition, *mpk6* proteome contained a higher number of proteins involved in seed germination as compared to *mpk4*. On the other hand, *mpk4* root proteome contained more proteins involved in seed dormancy and meristem maintenance.

Further, we manually selected differentially-regulated developmental proteins in both mutants. These proteins and their specific roles in plant development are listed in Tables 3 and 4.

**Figure 6:**
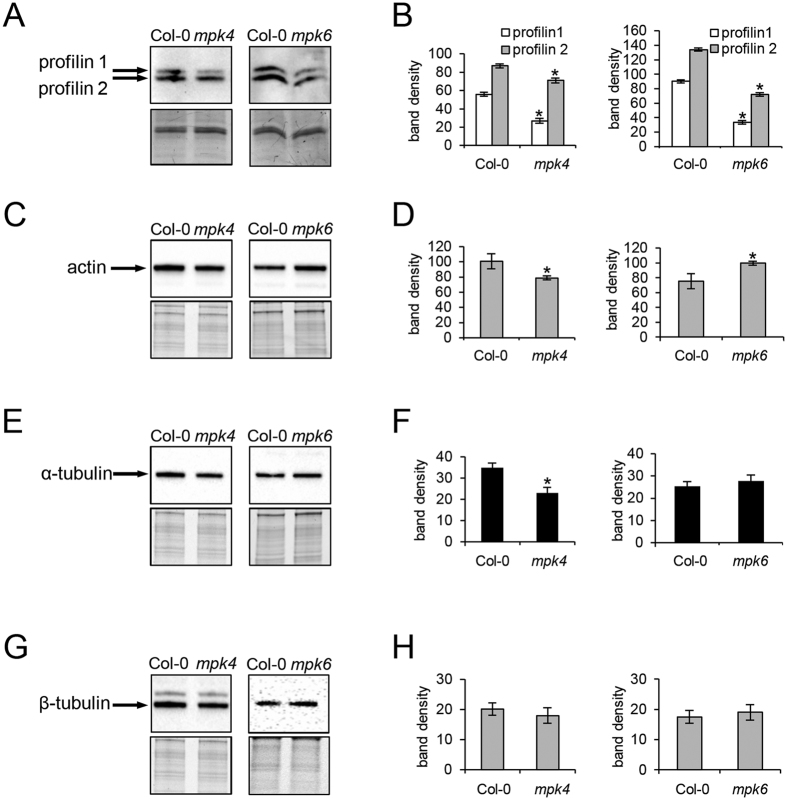
Immunoblotting differential analyses of profilins (**A,B**), actin (**C,D**), alfa-tubulin (**E,F**) and beta-tubulin (**G,H**) in the *mpk4* and *mpk6* mutants. Graphs depict optical density quantifications of relevant proteins. Loading controls are provided beneath the relevant immunoblots. Error bars are standard deviations calculated from 3 biological replicates. Stars indicate significant difference at p ≤ 0.05 according to Student *t*-test.

The cytoskeletal proteins are crucial regulators of cell division, elongation and growth. Both mutants are known for their altered organization of cortical and mitotic microtubules^12^,^13^,^14^. However, nothing is known about actin cytoskeleton in these mutants. Our results revealed changed abundances of several actin isoforms and actin binding proteins important for actin organization and dynamics in both *mpk4* and *mpk6* mutants. Thus, the *mpk4* mutant showed lower levels of profilin isoforms 1 and 2 (PRF1 and PRF2) when compared to the Col-0 wild type (Table 3). This was proven also by immunoblotting analysis using antibody recognizing both PRF1 and PRF2 (Fig. 6A,B). Profilin regulates actin polymerization while Arabidopsis *prf1* mutant plants display increased number of root hairs, a phenotype similar to the *mpk4*^39^. Dehydrin ERD10 has actin stabilizing activity^40^ and it was downregulated in the roots of the *mpk4* mutant. Moreover, annexin 1 showed more than four-fold higher abundance in this mutant. Annexins are stress induced proteins involved in actin filament bundling and in vesicular trafficking in plants^41^. Therefore, changed abundances of above-mentioned actin binding proteins suggest modifications of actin cytoskeleton organization in the *mpk4* mutant.

Actin cytoskeleton appeared to be altered also in the *mkp6* mutant, as indicated by the downregulation of actin 7 and profilin 2. On the other hand, actin 8 and actin-like ATPase superfamily protein were uniquely detected in the *mpk6* mutant and this might contribute to the regulation of actin cytoskeleton organization in the mutant. Moreover, increased actin abundance in the *mpk6* and decreased abundances of PRF1 and PRF2 were corroborated by immunoblotting using actin antibody recognizing all actin isoforms and profilin antibody recognizing PRF1 and PRF2 (Fig. 6A,B,C,D). Further, we detected increased abundance of alpha tubulin 5 and 6 in the *mpk6* mutant. Consistently, immunoblotting analysis showed slight overabundance of tubulin isoforms in the *mpk6* mutant, while lower abundances were encountered in the *mpk4* mutant (Fig. 6E–H). This analysis showed that abundances of tubulin isoforms were differently regulated in both mutants.

Phospholipase D alpha 1 (PLDa1), an enzyme producing phosphatidic acid (PA) and its lipid derivatives, was uniquely detected only in the *mpk6* mutant. PLDa1-derived PA binds to MAP65–1, enhances its activity during microtubule polymerization and bundling^42^. PA is capable also to activate MPK6^43^. Our proteomic data point to possible negative regulation of PLDa1 abundance by MPK6 and this might contribute to the altered cortical microtubule organization in the *mpk6* mutant^12^. PLDa1 is a putative candidate for MPK6-dependent phosphorylation as suggested by bioinformatic analysis (Table 2).

Additionally, several other proteins directly involved in cell division, a process controlled by both MPK6 and MPK4 12,14, were detected in both mutants (Tables 3 and 4). S-phase kinase-associated protein 1, subunit of an E3 ubiquitin ligase known as the SCF complex, was detected only in the *mpk4* mutant (Table 3), suggesting its increased abundance. This protein might act downstream of MPK4 in the regulation of cell division and meristem activity. Next, CDC-48A which localizes at the cell division plane during cytokinesis and contributes to membrane fusion events^44^ was overabundant in the *mpk6* mutant (Table 4). Thus it might be involved in ectopic and disoriented cell divisions of this mutant^12^.

Other proteins important for root growth and embryogenesis were predominantly downregulated in the *mpk4* mutant, consistently with its firmly reduced growth. For example, HSP90-like protein GRP94 (known also as SHEPERD) was five-fold upregulated (Table 3). This protein is important for CLAVATA receptor kinase folding and *shd* mutants show root apical meristem phenotype with disorganized columella cells and altered organization of initials and central cells^45^.

### Proteomics revealed differences in cellular compartment morphogenesis, endopeptidase activity and RNA processing between *mpk4* and *mpk6* mutants

Another GO annotation showing significant differences between *mpk4* and *mpk6* mutants in terms of biological process was cellular component organization (Fig. 2A). The *mpk6* mutant contained a higher number of proteins annotated to this class. Further evaluation showed that the *mpk6* differential proteome contained a higher number of proteins annotated to morphogenesis of cellular components, membrane organization, external encapsulating structure organization and organelle organization (Fig. 4C). Moreover, the *mpk6* differential proteome uniquely contained proteins involved in cellular component disassembly such as lipoxygenase 1 and glycine-rich RNA-binding protein 2.

**Figure 7:**
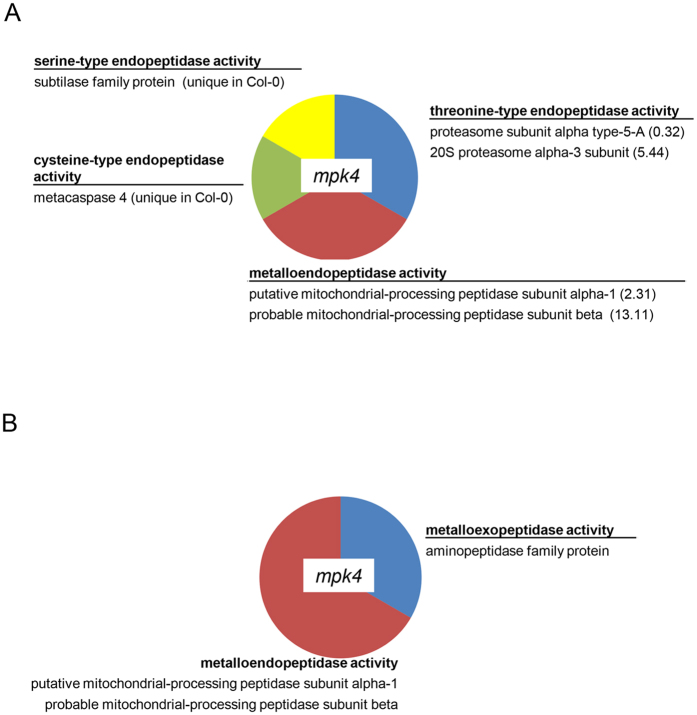
Graphs showing gene ontology categories related to (**A**) endopeptidase activity and (**B**) metalopeptidase activity in the *mpk4* mutant.

In terms of molecular function, root proteome of the *mpk4* mutant contained three GO annotations which were absent in the *mpk6* mutant (Fig. 2B). The *mpk4* mutant contained differentially regulated proteins with endopeptidase and metallopeptidase activity (Fig. 7A,B). Proteins with endopeptidase activity included proteases with different specificity depending on amino acids (Fig. 7A). This might indicate differences in proteolytic processes in the mutants.

Finally, proteomic analysis indicated decreased abundance of mRNA decapping complex VCS (known also as varicose) in the *mpk4* mutant. This protein is the component of the decapping complex, that removes the 7-methyl-guanosine 5′-diphosphate from the 5′ end of mRNAs. Decapping allows rapid changes in gene expression and is important for many stress related and developmental processes^46^. This protein contains the MAPK docking site and specific phosphorylation site in its amino acid sequence (Table 1) suggesting that it might be a putative substrate target of MPK6. Indeed, the MPK4-induced changes in VCS levels may contribute to deregulation of gene expression and resulting changed protein abundances in the *mpk4* mutant. Nevertheless, this hypothesis should be experimentally tested in future studies.

## Discussion

Considering versatility of MPK4 and MPK6 functions, OMICS technologies such as transcriptomics, proteomics and phosphoproteomics are powerful techniques to investigate different aspects of MAPK signalling^15^. They provide a wide range of information about transcripts and proteins being modified in MAPK mutants and help to identify new MAPK targets.

In this study, we aimed to decipher root proteomes of two Arabidopsis *mpk4* and *mpk6* mutants. Both MPK4 and MPK6 are known to be activated by oxidative stress and pathogens, however, they are likely involved in different MAPK signalling pathways^4^,^5^,^8^,^9^. Differential proteomic analysis of plants with genetically manipulated MAPKs could be useful for investigation of proteome-wide changes in such plants^15^.

### Identification of putative MPK4 and MPK6 targets

It was previously reported that MAPK knock-out mutants show changes in the transcriptional levels of some respective MAPK phosphorylation targets^47^. Using bioinformatic analysis we were able to predict several putative targets of MPK4 and MPK6 in analyzed proteomes. These were proteins of diverse localizations and functions and in some cases it corresponded well with known localization and function of MPK4 and MPK6. For example, CDC48A, which was detected only in the *mpk6* mutant is localized to the cell plate during cytokinesis, similarly to MPK6^48^,^49^. This indicated that CDC48A might be a putative target protein which is regulated by MPK6.

One protein candidate involved in gene transcription in the *mpk4* mutant is the mRNA decapping complex VCS which was detected only in the wild type plants (indicating its downregulation in the *mpk4* mutant). This protein might be, at least partially, responsible for MPK4-dependent transcriptomic and proteomic changes in Arabidopsis roots. mRNA decapping complex VCS was also found by phosphoproteomic study of transgenic plants expressing GVG:FLAG-NtMEK2^DD^ (constitutively active after dexamethasone treatment) as a putative MAPK target^50^. In this respect, two other proteins from our list, namely elongation factor EF2-like protein LOS1 and HSP 70–3 were also proposed as MAPK phosphorylation targets by protein microarray^51^.

### Distinct proteome composition of mpk4 and mpk6 mutants

Our proteomic analysis showed that knockout *MPK4* mutation affected broader range of proteins in Arabidopsis roots compared to *MPK6* knockout. A similar pattern was observed also in the recent transcriptomic study, which reported 1235 genes differentially expressed in the *mpk4* mutant but only 61 genes in the *mpk6* as compared to the Col-0^47^, although the difference in two mutant proteomes was not so pronounced as in the transcriptomic study. Nevertheless, it suggests that MPK4 has likely broader impact on the regulation of biological protein-dependent processes in Arabidopsis root when compared to MPK6. Next, GO annotation analysis showed functional categories unique either for *mpk4* or *mpk6* mutant differential proteomes. Proteins with peptidase activity as well as those involved in response to zinc, starvation and changing nutrient levels were affected in the *mpk4*, but not in the *mpk6* mutant. On the other hand, proteins involved in several developmental processes and cellular component disassembles were altered only in the *mpk6* mutant.

The relatively low number of differentially regulated proteins in *mpk6* mutant root implies that the absence of MPK6 might be compensated by MPK3, which is partially redundant to MPK6. Previously it was reported that MPK3 activities could be increased in the *mpk6* mutant in response flagellin in whole seedlings^47^ or hypoxia^52^ and ethylene in leaves^53^. However, little is known about basal abundance and activity of MPK3 in the *mpk6* mutant and even less emphasis was given on MPK3 levels and activity in resting mutant roots so far. Published data refer about small overlap in transcriptomic profiles of 2 weeks old *mpk3* and *mpk6* mutant seedlings under control conditions^47^ as well as leaf proteomes of 6 weeks old *mpk3* and *mpk6* mutants expressing MKK5^DD^ inducible by dexamethasone^54^.

Considering MPK3 abundance, no differences were found in the leaves of 5 weeks old *mpk6* mutant and wild type plants^55^. These data indicate that MPK3 might not compensate for MPK6 deficiency in the leaves of the *mpk6* mutant. In the 2 weeks old roots of the *mpk6* mutant, we have found higher abundance but lower activity of MPK3. Thus, the capability of MPK3 to compensate for MPK6 deficiency might depend on tissue and developmental stage of plants.

### The constitutive induction of pathogen defense is related to upregulation of ER body proteins in the *mpk4* mutant

Present proteomic analysis confirmed some known information about defense responses in Arabidopsis roots mediated by MPK4 and MPK6. For example, while MPK6 is a negative regulator of JA signalling^7^, MPK4 positively affects the JA signalling pathway^23^. This is fully consistent with elevated abundance of lipoxygenase 1, an enzyme crucial for JA synthesis, in the *mpk6* roots as well as with decreased abundance of jasmonic acid responsive protein 1 in *mpk4* roots found in our study.

MPK6 phosphorylates 1-aminocyclopropane-1-carboxylic acid synthase 2 and 6 leading to promotion of ethylene production^6^. We have found decreased abundance of 1-aminocyclopropane-1-carboxylate oxidase 2 in the *mpk6* mutant, suggesting additional level of ethylene regulation by MPK6.

On the other hand, abundances of pathogen related proteins were not fully consistent with constitutive activations of pathogen related proteins in the *mpk4* mutant^4^,^23^. We detected several ER-resident pathogenesis related proteins as upregulated in the mutant. These proteins are involved in ER body formation^56^,^57^, which is an important component of plant pathogen defense^22^. NAI2, beta glucosidase and JAL34 were identified in the *mpk4* mutant as upregulated, and they are controlled by transcription factor named NAI1. The analysis of MAPK-specific phosphorylation sites of NAI1 showed that MAPKs are most probable kinases responsible for phosphorylation of this transcription factor (Supplementary Table S9). NAI1 also contains docking motif for interaction with MAPK (Supplementary Table S10). All this implies that the constitutive activation of defense response in the *mpk4* mutant might be connected to ER body formation. Moreover, MAPKs such as MPK4 might be crucial regulators of ER body formation controlled by the phosphorylation of NAI1.

Still, several proteins involved in pathogen defense were downregulated in the *mpk4* mutant. This could be perhaps explained by the selective activation of the pathogen defense by MPK4, depending on leucine-rich receptors^58^.

### MPK4 is important for antioxidant defense

Members of MAPK cascades are important mediators of antioxidant defense in plants. This was shown for MKK5 in high-light induced oxidative stress for Cu/Zn SOD^59^ and salinity induced FeSOD^60^ as well as for MKK1 and MPK6 in the regulation of CAT1^61^. Here we report, that roots of *mpk4* mutant possess increased levels of some proteins and enzymes involved in antioxidant defense. Similarly, increased levels of transcripts of antioxidant genes were obtained in transcriptomic study on seedlings^9^. Such increased antioxidant defense correlates with increased levels of ROS in the *mpk4* mutant^4^. When compared to *mpk4*, *mpk6* roots showed decreased abundance of monodehydroascorbate reductase.

Since these proteomic data revealed that the antioxidant defense might be differentially regulated by these two kinases, we aimed to examine enzymatic activities of peroxidase and catalase decomposing hydrogen peroxide. Our results showed that mainly catalase activity was significantly upregulated in the *mpk4* mutant, unlikely to the *mpk6* mutant. Previously we reported that constitutive upregulation of antioxidant defense caused increased tolerance of Arabidopsis *anp2anp3* double mutant to the oxidative stress^62^. Similarly, our present data point to possible negative regulation of antioxidant defense by MPK4.

### Proteomic analysis deciphering mpk4 and mpk6 root phenotypes

Both mutants have distinct phenotypes. The *mpk4* mutant shows severely reduced growth^23^ and short roots with radial expansion and strong root hair defects^13^. At the subcellular level, the *mpk4* mutant has cytokinetic defects resulting from various abnormalities in mitotic spindle and phragmoplast rearrangements^14^. Knockout *mpk6* mutant displays “no root”, “short root” or “long root” phenotypes at early seedling development and increased number of adventitious roots at the later developmental stages, as well as ectopic cell divisions and defective cell plate orientation^12^,^66^.

In fact, some proteins directly involved in cell division were altered in both mutants. CDC-48A protein, a potential MPK6 phosphorylation target (see discussion above), which contributes to cell division plate formation^44^, showed overabundance in the *mpk6* mutant. Since MPK6 localizes in spots close to cell plate and determines the cell plane orientation^12^ these data might indicate a negative regulation of CDC-48A by MPK6 during cell plate formation.

Concerning actin cytoskeleton, both mutants showed decreased abundance of profilins PRF1 and PRF2. Profilin is an actin binding protein crucial for root hair formation^63^, a process which is severely altered in the *mpk4* mutant^13^. Plant profilins regulate actin polymerization dynamics and filament initiation together with other actin-binding proteins (ABPs) such as actin depolymerizing factors (ADFs), actin-related proteins (ARPs) and formins. Depending on the equilibrium between these ABPs actin polymerization might be either promoted or inhibited^64^. Since actin polymerization and dynamics is important for cell and organ elongation growth–hypothetically, less profilins and less actin might be connected with shorter roots in the *mpk4* mutant while less profilin but more actin and ADF might result in dominating long-root phenotype in the *mpk6* mutant.

In conclusion, present proteomic study identified several protein candidates contributing to the phenotypes and defense responses in the Arabidopsis *mpk4* and *mpk6* mutants, and paved the way for their functional characterization in the future studies.

## Materials and Methods

### Material

Seeds of *Arabidopsis thaliana,* wild type (ecotype Col-0), *mpk4*^13^ and *mpk6–2*^12^ mutants were grown vertically on Petri dishes with 1/2 Murashige-Skoog solid culture medium^65^ (pH 5.7) (16 h light/8 h dark, 22 °C). For *mpk6–2* mutant, seedlings displaying “short root” and dominating “long root” phenotypes^66^ were used for the analyses. Roots were collected for proteomic and biochemical analyses two weeks after germination. Proteomic analyses were performed on four independent biological replicates.

### Protein extraction for proteomic analysis

Proteins were extracted as described previously^67^. Roots of Arabidopsis wild type plants and mutants were homogenized in liquid nitrogen to fine powder and extracted in buffer containing 0.9 M sucrose, 0.1 M Tris-HCl, pH 8.8, 10 mM EDTA, 100 mM KCl and 0.4% v/v 2-mercaptoethanol. Total proteins were obtained from the extract using phenol extraction followed by precipitation in methanolic ammonium acetate (100 mM), and sequential purification in 80% v/v acetone, 70% v/v ethanol, and final incubation with 80% v/v acetone. The final protein pellet was dissolved in 6 M urea in 100 mM Tris-HCl, pH 6.8. Proteins were reduced and alkylated prior to trypsin digestion (1 μg of trypsin was applied to 50 μg of proteins). Digested peptides were desalted on C18 cartridges (Sep PAK, Waters, Milford, MA, USA) and vacuum dried.

### Liquid Chromatography and Mass Spectrometry

Spectral data were collected using an Orbitrap LTQ Velos mass spectrometer (Thermo Fisher Scientific, Waltham, MA, USA) using Xcalibur version 2.1.0 united with an UltiMate 3000 nano flow HPLC system (Dionex). Two μg of protein tryptic digest were loaded on reversed phase fused silica C18 column measuring 75 μm × 150 mm (Thermo Fisher Scientific). Peptides were separated and eluted at a constant flow rate of 0.3 μl.min-1 by a 170-minute long nonlinear gradient of acetonitrile (in 0.1% formic acid) as follows: 2–55% for 125 min, 95% for 15 min, 5% for 30 min. Peptides were detected in linear trap mass spectrometer operated in a data dependent acquisition (DDA) mode. The mass spectra were obtained in the data dependent mode, with dynamic exclusion being applied, in 18 scan events: one MS scan (m/z range: 300–1700) followed by 17 MSMS scans for the 17 most intense ions detected in the MS scan. Other critical parameters were set as follows: Normalized collision energy: 35%, AGC (automatic gain control) “on” with MSn Target 4 × 104, isolation width (m/z): 1.5, capillary temperature 170 °C, spray voltage 1.97 kV.

The .raw files were searched using the SEQUEST algorithm of the Proteome Discoverer 1.1.0 software (Thermo Fisher Scientific) with following selection of parameters: Lowest and highest charge: +1 and +3, respectively; minimum and maximum precursor mass: 300 and 6000 Da, respectively; minimum S/N ratio: 3; enzyme: trypsin; maximum missed cleavages: 2, FDR = 0.01; dynamic modifications: cysteine carbamidomethylation (+57.021), methionine oxidation (+15.995), methionine dioxidation (+31.990).

The spectral data were matched against target and decoy databases (later being created automatically by the software). The NCBI (www.ncbi.nlm.nih.gov) Arabidopsis genus taxonomy referenced protein database (67,924 entries as of November 2013) served as the target database, while its reversed copy served as a decoy database. The Proteome Discoverer results files (.msf) were uploaded to ProteoIQ 2.1 (NuSep) software for further filtering. Only proteins detected with at least three spectral counts, FDR < 1%, 95% probability and listed as “Top” proteins (defined by ProteoIQ, “Within a protein group, each and every respective peptide could be matched to the top protein”) are considered as high confidence matches and are presented in the results. The relative quantitative analysis was based on sums of precursor ion intensities of filtered peptides attributed to given proteins. The ANOVA analysis of four replicates for each biological sample was performed and p ≤ 0.05 was used to filter statistically significant results.

### Bioinformatic evaluation of proteomic data

Proteins with statistically significantly changed abundances as well as those detected uniquely in one of the two analyzed samples were subjected to gene ontology annotation using Blast2Go^17^ software. The full sequences retrieved from NCBI database were blasted against Plants/*Arabidopsis thaliana* protein sequences allowing 1 BLAST Hit, followed by mapping step and annotation by using these parameters: E Value Hit filter: 1.0E-6; Annotation cut off: 55; GO weight: 5. One GO level was used for the evaluation of GO annotations according to molecular function, biological process and compartment.

Amino acid sequences of proteins with statistically significantly changed abundances as well as those detected uniquely in one of the two analyzed samples were screened for the presence of MAPK specific docking domains using Eukaryotic Linear Motif (ELM) resource^68^. The identified proteins were further screened for the presence of MAPK-specific phosphorylation motif by using GPS 3.0–Kinase-specific Phosphorylation Site Prediction^69^. Finally, Wolf Psort prediction tool (http://www.genscript.com/wolf-psort.html) was used for the prediction of protein subcellular localization.

### Immunoblotting analysis of actin, tubulin and profilin abundances in MAPK mutants

Protein extracts obtained by phenol extraction were enriched with SDS sample buffer and 2-mercaptoethanol (5% v/v) and used for immunoblotting analysis. For SDS-PAGE, Stain Free^TM^ technology (Biorad, Hercules, CA, USA) was applied allowing UV-based visualization of proteins on gel and membrane to ensure equal sample loading. Identical protein amounts were loaded for each sample. Proteins were transferred to a polyvinylidene difluoride (PVDF) membrane (GE Healthcare, Little Chalfont, United Kingdom) in a wet tank unit (Biorad, Hercules, CA, USA) at 100 V for 1.5 h. For immuno-detection of protein bands, the membrane was blocked in a mixture of 4% w/v low-fat dry milk and 4%w/v bovine serum albumin in Tris-buffered-saline (TBS, 100 mM Tris-HCl; 150 mM NaCl; pH 7.4) for 1 hour, and subsequently incubated with anti-profilin, anti-beta tubulin, anti-actin (Sigma-Aldrich, Germany), and anti-alpha tubulin (Abd Serotec, Kidlington, UK) at room temperature for 1.5 h. After it, they were incubated with a horseradish peroxidase conjugated goat anti-rabbit IgG secondary antibody (Santa Cruz Biotechnology, Santa Cruz, CA, USA), diluted 1:5000 in TBS-T containing 1% w/v BSA at room temperature for 1.5 h. Following five washing steps in TBST, proteins were detected by incubating the membrane in Clarity Western ECL substrate (Biorad, Hercules, CA, USA). Luminescence was detected using Chemidoc MP documentation system (Biorad). Immunoblot analyses were performed in three biological replicates. The band signal density was quantified using Image Lab 4.0.1 software (Biorad, Hercules, CA, USA). The differences between the mutants and the wild type were statistically evaluated using Students t-test (n = 3).

### Immunoblotting analysis of MPK3 and MPK6 activities

For the examination of MPK3 activity in Col-0 and *mpk6* mutant roots, we extracted the proteins in E buffer (50 mM HEPES (pH 7.5), 75 mM NaCl, 1 mM EGTA, 1 mM MgCl_2_, 1 mM NaF,10% v/v glycerol, Complete™ EDTA-free protease inhibitor and PhosSTOP™ phosphatase inhibitor cocktails (both from Roche, Basel, Switzerland). Following centrifugation, equal protein aliquots were subjected to overnight precipitation by addition of 5 volumes of 80% ice cold acetone. The resulting precipitates were dissolved in SDS sample buffer and boiled at 95 °C for 5 minutes. Immunoblotting was performed as described above, using polyclonal antibody against mammalian phosphorylated ERK1/2 (phospho-p44/42, pERK; Cell Signaling; Danvers, ME, USA). The differences between the mutants and the wild type were statistically evaluated using Students t-test (n = 3).

### Analysis of enzymatic activities

For the assessment of enzymatic activities in untreated plants, we harvested the roots of the wild type Col-0 plants as well as *mpk4 and mpk6* mutants grown 14 days on solid ½ MS. For the examination of enzymatic activities in response to oxidative stress treatment, 14 days old seedlings were incubated either in liquid ½ MS medium, or in the same medium supplemented with 150 μM H_2_O_2_ for 24 h. For preparation of native protein extract, roots were homogenized in liquid nitrogen and the homogenate was incubated with 50 mM sodium phosphate buffer (pH 7.8) containing 1 mM EDTA, 10% v/v glycerol and “Complete EDTA-free protease inhibitor cocktail (Roche, Basel, Switzerland). Following centrifugation at 13000 g at 4 °C, protein content was estimated in supernatants using Bradford assay^70^. Equal protein amounts were used for further analyses. Isozymes of peroxidases and catalase were separated on 12% native PAGE at constant 10 mA/gel. For visualization of peroxidases, gels were first equilibrated in 50 mM sodium acetate (pH 5,2) buffer for 15 min, followed by incubation in 0.05% (w/v) o-dianizidine and 3% (v/v) hydrogen peroxide in 50 mM sodium acetate (pH 5.2). Catalase isozyme was visualized according to Aebi^71^. Gels were three times washed in distilled water for 5 minutes followed by incubation in 0.006% (v/v) hydrogen peroxide for 10 min. Catalase activity appeared as negative bands after 5 min of gel incubation in 1% (w/v) potassium ferricyanide and 1% (w/v) ferric chloride in distilled water. The band intensities were calculated using Image Lab 4.0.1 software (Biorad). Analyses were performed in biological triplicates. Statistical evaluation of data was carried out using Student’s t-test. As a loading control equal amounts of protein were resolved by SDS-PAGE as described above with the exception that precast 4–12% gradient gels (Mini-PROTEAN TGX Stain-Free Precast Gels, BioRad) were used allowing UV-induced total protein visualization with Chemidoc MP imaging system (BioRad). All experiments were carried out in biological triplicates.

## Additional Information

**How to cite this article**: Takáč, T. *et al.* Comparative proteomic study of Arabidopsis mutants *mpk4* and *mpk6. Sci. Rep.* **6**, 28306; doi: 10.1038/srep28306 (2016).

## Supplementary Material

Supplementary Information

## Figures and Tables

**Table 1:** List of differentially regulated proteins containing both MAPK-specific phosphorylation site and MAPK docking site as predicted by GPS 3.0 software and Eukaryotic Linear Motif Resource (http://elm.eu.org/index.html) in the roots of the *mpk4* mutant.

Accesion number	Protein name	Phosphopeptide sequences	Docking sequence	Fold change	Cell compartment
gi|145324054	arabinogalactan protein 31	EVNHKTQTPSLAPAP	KFNRSLVAV, 180–188 [A]	unique in wild type	cell wall^72^
		HPHPPAKSPVKPPVK	KKLGKSTVVV, 285–294 [A]		
		PPVKAPVSPPAKPPV	KLGKSTVVV, 286–294 [A]		
		PPVKPPVSPPAKPPV			
		APVKPPVSPPTKPPV			
		PPTKPPVTPPVYPPK			
gi|15226573	Ferredoxin–nitrite reductase	SFSLTFTSPLLPSSS	KPKRSVLV, 19–26 [A]	unique in wild type	plastid (prediction)
			KIEREPMKL, 74–82 [A]		
			KSSKDDIDVRL, 103–113 [A]		
			RKWNVCV, 246–252 [A]		
			KDGRFGFNLLV, 274–284 [A]		
			KRCEEAIPL, 291–299 [A]		
			RQKTRMMWL, 328–336 [A]		
			KKGVRVTELVPL, 554–565 [A]		
			KGVRVTELVPL, 555–565 [A]		
gi|15233349	aconitate hydratase 1	RATIANMSPEYGATM	RIDKLPYSIRI, 35–45 [A]	unique in wild type	cytosol, mitochondria^73^
		YFKGMTMSPPGPHGV	KRPHDRVPL, 378–386 [A]		
			KKACDLGL, 458–465 [A]		
gi|18400212	dihydrolipoamide acetyltransferase, long form protein	TTSTKLSSPMAGPKL	RRDHAVAV, 20–27 [A]	unique in wild type	mitochondria^74^
		EIGMPSLSPTMTEGN			
gi|22331076	Subtilase family protein	PLLLCFFSPSSSSSD	RRHPSVISV, 93–101 [A]	unique in wild type	extracellular (prediction)
		LLRSLPSSPQPATLL			
		HGFSARLSPIQTAAL			
		REIHTTHTPAFLGFS			
		LGTLIGPSPPSPRVA			
		LIGPSPPSPRVAAFS			
		ANVEIDVSPSKLAFS			
gi|240254562	uncharacterized protein	SSSGNVTTPTQTAST	KSRDIDLSF, 1264–1272 [A]	unique in wild type	nucleus (prediction)
gi|30682607	mRNA decapping complex VCS	PGISAQPSPVTQQQQ	RKAQPLVVL, 352–360 [A]	unique in wild type	cytoplasmic foci^75^
		TPPLNLQSPRSNHNP	KESKRLEVAL, 918–927 [A]		
		TLPQLPLSPRLSSKL	KRLEVAL, 921–927 [A]		
gi|15232845	probable mitochondrial-processing peptidase subunit beta	DSVPASASPTALSPP	RRSQRRLFL, 11–19 [A]	13.11	mitochondria
		SASPTALSPPPPHLM	RINRERDVIL, 210–219 [A]		
gi|15221019	GDSL esterase/lipase	ITVAGQNSPVVALFT			extracellular (prediction)
		KFSDGLITPDFLAKF	KFMKIPLAI, 88–96 [A]	5.48	
		REFWVPPTPATVHAS			
gi|15232603	60S acidic ribosomal protein P0-2	KGTVEIITPVELIKQ	RKGLRGDSVVL, 44–54 [A]		ribosome
		YDNGSVFSPEVLDLT	KGLRGDSVVL, 45–54 [A]	4.61	
			KINKGTVEI, 148–156 [A]		
gi|186513287	argininosuccinate synthase	ALNGKALSPATLLAE	RGKLKKVVL, 93–101 [A]		plastid^76^
			KKHNVPVPV, 255–263 [A]	3.63	
			KKDMYMMSV, 293–301 [A]		
			KLYKGSVSV, 393–401 [A]		
gi|15240765	voltage dependent anion channel 2	DITATLGSPVISFGA	KHPRFGLSLAL, 264–274 [A]	2.41	mitochondria, plasma membrane^24^
gi|15218090	putative mitochondrial-processing peptidase subunit alpha-1	VAETSSSTPAYLSWL			mitochondria
		LKIASETTPNPAASI	RKMKVEI, 196–202 [A]	2.31	
		GYSGPLASPLYAPES			
gi|334185190	heat shock protein 70-3	DLLLLDVTPLSLGLE	RARFEELNI, 305–313 [A]	0.57	nucleus, cytoplasm^30^
			RIPKVQQLLV, 348–357 [A]		
gi|14532542	proteasome subunit alpha type-5-A	AVEKRITSPLLEPSS	KTKEGVVLAV, 41–50 [A]	0.32	peroxisome, cytoplasm (prediction)
gi|30691626	heat shock protein 70-1	GTSGTEQTPEAEFEE	KRSDNIDL, 289–296 [A]	0.32	cytosol^77^
			KKQLIDL, 573–579 [A]		
gi|30696056	elongation factor EF-2	EEMQRPGTPLYNIKA	KRLAKSDPMVV, 509–519 [A]	0.08	cytoplasm^78^
		KGLKEAMTPLSEFED	RLAKSDPMVV, 510–519 [A]		

**Table 2:** List of differentially regulated proteins containing both MAPK-specific phosphorylation site and MAPK docking site as predicted by GPS 3.0 software and Eukaryotic Linear Motif Resource (http://elm.eu.org/index.html) in the roots of the *mpk6* mutant.

Accesion number	Protein name	Phosphopeptide sequences	Docking sequence	Fold change	Cell compartment
gi|15230005	regulatory particle triple-A ATPase 5A	*****MATPMVEDTS	RKGKCVVL, 102–109 [A]	unique in *mpk6*	proteasome
			KERFEKLGV, 194–202 [A]		
gi|15232671	phospholipase D alpha 1	AAAGFPESPEAAAEA	RRPKPGGDVTI, 244–254 [A]	unique in *mpk6*	plasma membrane^79^, cytoplasm, nucleus (prediction)
			RPKPGGDVTI, 245–254 [A]		
			KKKASEGVRV, 259–268 [A]		
			KKASEGVRV, 260–268 [A]		
			KLRDLSDIII, 439–448 [A]		
			RRAKDFIYV, 511–519 [A]		
			RAKDFIYV, 512–519 [A]		
			KGEKFRVYVVV, 561–571 [A]		
			KFRVYVVV, 564–571 [A]		
gi|15232776	cell division control protein 48-A	*****MSTPAESSDS	RKKSPNRLVV, 23–32 [A]	unique in *mpk6*	nucleus, cytoplasm^80^
			KKSPNRLVV, 24–32 [A]		
			KVVRSNLRVRL, 90–100 [A]		
			RVRLGDVISV, 97–106 [A]		
			RPVRKGDLFL, 148–157 [A]		
			RKGDLFL, 151–157 [A]		
			KSRAHVIV, 339–346 [A]		
			RRFGRFDREIDI, 361–372 [A]		
			RFGRFDREIDI, 362–372 [A]		
			RFDREIDIGV, 365–374 [A]		
			KNMKLAEDVDL, 389–399 [A]		
			REKMDVIDL, 427–435 [A]		
			RPGRLDQLIYI, 639–649 [A]		
gi|15233111	cysteine synthase C1	AYDLLDSTPDAFMCQ	KRDASLLI, 50–57 [A]	unique in *mpk6*	plastids, cytosol, mitochondria^81^
			KSKNPNVKI, 238–246 [A]		
			KGKLIVTI, 331–338 [A]		
gi|334186086	ketol-acid reductoisomerase	APSLSCPSPSSSSKT	KKEKVSL, 88–94 [A]	3.44	plastid (prediction)
		GWSVALGSPFTFATT			
gi|334185190	heat shock protein 70-3	DLLLLDVTPLSLGLE	RARFEELNI, 305–313 [A]	3.15	nucleus, cytoplasm^30^
			RIPKVQQLLV, 348–357 [A]		
gi|145324054	arabinogalactan protein 31	EVNHKTQTPSLAPAP	KFNRSLVAV, 180–188 [A]	0.64	cel wall^72^
		HPHPPAKSPVKPPVK	KKLGKSTVVV, 285–294 [A]		
		PPVKAPVSPPAKPPV	KLGKSTVVV, 286–294 [A]		
		PPVKPPVSPPAKPPV			
		APVKPPVSPPTKPPV			
		PPTKPPVTPPVYPPK			
gi|15221156	pyrophosphate–fructose-6-phosphate 1-phosphotransferase subunit beta 1	RDLTAVGSPENAPAK	KKAMVEL, 512–518 [A]	0.08	plastid, cytoplasm (prediction)

**Table 3:** Differentially abundant proteins in the *mpk4* involved in the plant development as classified according to GO annotations.

Accession	Protein name	Fold change	Developmental process
gi|18417863	14-3-3-like protein gf14 upsilon	unique in WT	root formation, chloroplast development^82^
gi|30682607	mRNA decapping complex vcs	unique in WT	leaf blade development^83^; early seedling development^46^
gi|30695409	acetoacetyl- thiolase 2	unique in WT	pollen tube elongation, embryogenesis^84^
gi|15219345	metacaspase 4	unique in WT	embryogenesis^85^
gi|15224838	profilin 1	0.36	root hair formation^63^
gi|15233538	profilin 2	0.25	root hair formation^63^
gi|18379240	mlp-like protein 328	0.35	bolting^86^
gi|15231255	tcp-1 cpn60 chaperonin family protein	0.23	plastid division^87^
gi|145329204	triosephosphate isomerase	0.45	transition from heterotrophic to autotrophic growth^88^
gi|15234781	peptidyl-prolyl cis-trans isomerase cyp1	0.54	stem elongation and shoot branching^89^
gi|15237054	v-type proton atpase subunit e1	0.11	embryo development^90^
gi|145323784	l-ascorbate peroxidase 1	0.24	embryogenesis^91^
gi|30686836	dehydrin erd10	0.32	seed development^92^
gi|145333043	adenosylhomocysteinase 1	25.25	seed and root development^93^
gi|18410982	s-phase kinase-associated protein 1	*mpk4* unique	cell division, meiosis^94^,^95^, meristem activity^96^, seedling development^97^
gi|15233740	hsp90-like protein GRP94	4.99	shoot, floral and root meristem function^45^

Only proteins with published experimental evidence for their role in the plant development are listed here.

**Table 4:** Differentially abundant proteins in the *mpk6* involved in the plant development as classified according to GO annotations.

Accession	Protein name	Fold change	Developmental process
gi|145333041	glycine-rich RNA-binding protein 2	0.136	seedling development and germination^98^, flower and seed development^99^
gi|15220770	1-aminocyclopropane-1-carboxylate oxidase 2	0.094	ethylene synthesis^71^, seed germination^100^,^101^
gi|15231569	aquaporin tip1-2	0.077	lateral root emergence^102^
gi|15228041	aquaporin tip1-1	0.053	lateral root emergence^102^
gi15233538	profilin 2	0.209	root hair formation^63^
gi|30691988	chaperone protein dnaj 3	0.136	flowering^103^
gi|18403295	gamma-aminobutyrate transaminase pop2	0.139	cell wall composition, root and hypocotyl development^104^
gi|15229522	adenosylhomocysteinase 2	1.594	seed and root development^93^
gi|15222075	actin 8	unique in *mpk6*	root hair tip growth^105^
gi|30683070	tubulins	5.012	helical root growth^106^, gravitropism^107^
gi|15242516	actin 7	5.805	callus formation^108^; seed germination and root growth^109^, cell division^105^
gi|15232671	phospholipase d alpha 1	unique in *mpk6*	stomatal closure^110^
gi|15220941	guanine nucleotide-binding protein subunit beta-like protein a	unique in *mpk6*	root formation, seed germination and seedling development^37^,^111^
gi|15221970	lipoxygenase 1	unique in *mpk6*	formation of lateral roots^112^
gi|15230005	regulatory particle triple-a atpase 5a	unique in *mpk6*	gametophyte development^113^, root apical meristem maintenance^114^
gi|15232776	cell division control protein 48-a	unique in *mpk6*	cell division, expansion and differentiation^48^
gi|15233320	aquaporin tip2-1	unique in *mpk6*	lateral root emergence^102^
gi|30691619	elongation factor 1b beta	unique in *mpk6*	cell wall formation and cell expansion^115^

Only proteins with published experimental evidence for their role in the plant development are listed here.

## References

[b1] Smékalová V., Doskočilová A., Komis G. & Šamaj J. Crosstalk between secondary messengers, hormones and MAPK modules during abiotic stress signalling in plants. Biotechnol. Adv. 32, 2–11 (2014). 23911976 10.1016/j.biotechadv.2013.07.009

[b2] Colcombet J. & Hirt H. Arabidopsis MAPKs: a complex signalling network involved in multiple biological processes. Biochem. J. 413, 217–226 (2008). 18570633 10.1042/BJ20080625

[b3] Teige M. *et al.* The MKK2 Pathway Mediates Cold and Salt Stress Signaling in Arabidopsis. Mol. Cell 15, 141–152 (2004). 15225555 10.1016/j.molcel.2004.06.023

[b4] Gao M. *et al.* MEKK1, MKK1/MKK2 and MPK4 function together in a mitogen-activated protein kinase cascade to regulate innate immunity in plants. Cell Res. 18, 1190–1198 (2008). 18982020 10.1038/cr.2008.300

[b5] Asai T. *et al.* MAP kinase signalling cascade in Arabidopsis innate immunity. Nature 415, 977–983 (2002). 11875555 10.1038/415977a

[b6] Liu Y. & Zhang S. Phosphorylation of 1-aminocyclopropane-1-carboxylic acid synthase by MPK6, a stress-responsive mitogen-activated protein kinase, induces ethylene biosynthesis in Arabidopsis. Plant Cell 16, 3386–3399 (2004). 15539472 10.1105/tpc.104.026609 PMC535880

[b7] Takahashi F. *et al.* The Mitogen-Activated Protein Kinase Cascade MKK3–MPK6 Is an Important Part of the Jasmonate Signal Transduction Pathway in Arabidopsis. Plant Cell 19, 805–818 (2007). 17369371 10.1105/tpc.106.046581 PMC1867372

[b8] Kovtun Y., Chiu W. L., Tena G. & Sheen J. Functional analysis of oxidative stress-activated mitogen-activated protein kinase cascade in plants. Proc. Natl. Acad. Sci. USA 97, 2940–2945 (2000). 10717008 10.1073/pnas.97.6.2940 PMC16034

[b9] Pitzschke A., Djamei A., Bitton F. & Hirt H. A major role of the MEKK1-MKK1/2-MPK4 pathway in ROS signalling. Mol. Plant 2, 120–137 (2009). 19529823 10.1093/mp/ssn079 PMC2639734

[b10] Bush S. M. & Krysan P. J. Mutational evidence that the Arabidopsis MAP kinase MPK6 is involved in anther, inflorescence, and embryo development. J. Exp. Bot. 58, 2181–2191 (2007). 17519351 10.1093/jxb/erm092

[b11] Bergmann D. C., Lukowitz W. & Somerville C. R. Stomatal Development and Pattern Controlled by a MAPKK Kinase. Science 304, 1494–1497 (2004). 15178800 10.1126/science.1096014

[b12] Müller J. *et al.* Arabidopsis MPK6 is involved in cell division plane control during early root development, and localizes to the pre-prophase band, phragmoplast, *trans*-Golgi network and plasma membrane. Plant J. Cell Mol. Biol. 61, 234–248 (2010). 10.1111/j.1365-313X.2009.04046.x 19832943

[b13] Beck M., Komis G., Muller J., Menzel D. & Šamaj J. Arabidopsis Homologs of Nucleus- and Phragmoplast-Localized Kinase 2 and 3 and Mitogen-Activated Protein Kinase 4 Are Essential for Microtubule Organization. Plant Cell 22, 755–771 (2010). 20215588 10.1105/tpc.109.071746 PMC2861451

[b14] Beck M., Komis G., Ziemann A., Menzel D. & Šamaj J. Mitogen-activated protein kinase 4 is involved in the regulation of mitotic and cytokinetic microtubule transitions in *Arabidopsis thaliana*. New Phytol. 189, 1069–1083 (2011). 21155826 10.1111/j.1469-8137.2010.03565.x

[b15] Takáč T. & Šamaj J. Advantages and limitations of shot-gun proteomic analyses on Arabidopsis plants with altered MAPK signaling. Plant Proteomics 6, 107 (2015). 10.3389/fpls.2015.00107 PMC4340173 25763005

[b16] Jensen L. J. *et al.* STRING 8–a global view on proteins and their functional interactions in 630 organisms. Nucleic Acids Res. 37, D412–416 (2009). 18940858 10.1093/nar/gkn760 PMC2686466

[b17] Conesa A. & Götz S. Blast2GO: A comprehensive suite for functional analysis in plant genomics. Int. J. Plant Genomics 2008, 619832 (2008). 18483572 10.1155/2008/619832 PMC2375974

[b18] Hord C. L. H. *et al.* Regulation of Arabidopsis early anther development by the mitogen-activated protein kinases, MPK3 and MPK6, and the ERECTA and related receptor-like kinases. Mol. Plant 1, 645–658 (2008). 19825569 10.1093/mp/ssn029

[b19] Kosetsu K. *et al.* The MAP kinase MPK4 is required for cytokinesis in *Arabidopsis thaliana*. Plant Cell 22, 3778–3790 (2010). 21098735 10.1105/tpc.110.077164 PMC3015120

[b20] Meng X. & Zhang S. MAPK Cascades in Plant Disease Resistance Signaling. Annu. Rev. Phytopathol. 51, 245–266 (2013). 23663002 10.1146/annurev-phyto-082712-102314

[b21] Doxey A. C., Yaish M. W. F., Moffatt B. A., Griffith M. & McConkey B. J. Functional Divergence in the Arabidopsis β-1,3-Glucanase Gene Family Inferred by Phylogenetic Reconstruction of Expression States. Mol. Biol. Evol. 24, 1045–1055 (2007). 17272678 10.1093/molbev/msm024

[b22] Yamada K., Hara-Nishimura I. & Nishimura M. Unique Defense Strategy by the Endoplasmic Reticulum Body in Plants. Plant Cell Physiol. 52, 2039–2049 (2011). 22102697 10.1093/pcp/pcr156

[b23] Petersen M. *et al.* Arabidopsis MAP Kinase 4 Negatively Regulates Systemic Acquired Resistance. Cell 103, 1111–1120 (2000). 11163186 10.1016/s0092-8674(00)00213-0

[b24] Lee S. M. *et al.* Pathogen inducible voltage-dependent anion channel (AtVDAC) isoforms are localized to mitochondria membrane in Arabidopsis. Mol. Cells 27, 321–327 (2009). 19326079 10.1007/s10059-009-0041-z

[b25] Hwang S.-G. *et al.* The Arabidopsis short-chain dehydrogenase/reductase 3, an ABSCISIC ACID DEFICIENT 2 homolog, is involved in plant defense responses but not in ABA biosynthesis. Plant Physiol. Biochem. 51, 63–73 (2012). 22153241 10.1016/j.plaphy.2011.10.013

[b26] Li J., Brader G. & Palva E. T. Kunitz trypsin inhibitor: an antagonist of cell death triggered by phytopathogens and fumonisin b1 in Arabidopsis. Mol. Plant 1, 482–495 (2008). 19825555 10.1093/mp/ssn013

[b27] Perl-Treves R., Foley R. C., Chen W. & Singh K. B. Early induction of the Arabidopsis GSTF8 promoter by specific strains of the fungal pathogen *Rhizoctonia solani*. Mol. Plant-Microbe Interact. MPMI 17, 70–80 (2004). 14714870 10.1094/MPMI.2004.17.1.70

[b28] Park S.-C. *et al.* Characterization of a heat-stable protein with antimicrobial activity from *Arabidopsis thaliana*. Biochem. Biophys. Res. Commun. 362, 562–567 (2007). 17720140 10.1016/j.bbrc.2007.07.188

[b29] Fujisaki K. & Ishikawa M. Identification of an *Arabidopsis thaliana* protein that binds to tomato mosaic virus genomic RNA and inhibits its multiplication. Virology 380, 402–411 (2008). 18762309 10.1016/j.virol.2008.07.033

[b30] Dufresne P. J. *et al.* Heat shock 70 protein interaction with Turnip mosaic virus RNA-dependent RNA polymerase within virus-induced membrane vesicles. Virology 374, 217–227 (2008). 18222516 10.1016/j.virol.2007.12.014

[b31] Noël L. D. *et al.* Interaction between SGT1 and cytosolic/nuclear HSC70 chaperones regulates Arabidopsis immune responses. Plant Cell 19, 4061–4076 (2007). 18065690 10.1105/tpc.107.051896 PMC2217652

[b32] Sun Q.-P., Guo Y., Sun Y., Sun D.-Y. & Wang X.-J. Influx of extracellular Ca2+ involved in jasmonic-acid-induced elevation of [Ca2+]cyt and JR1 expression in *Arabidopsis thaliana*. J. Plant Res. 119, 343–350 (2006). 16708291 10.1007/s10265-006-0279-x

[b33] He Y., Fukushige H., Hildebrand D. F. & Gan S. Evidence Supporting a Role of Jasmonic Acid in Arabidopsis Leaf Senescence. Plant Physiol. 128, 876–884 (2002). 11891244 10.1104/pp.010843 PMC152201

[b34] Voll L. M. *et al.* Loss of cytosolic NADP-malic enzyme 2 in *Arabidopsis thaliana* is associated with enhanced susceptibility to Colletotrichum higginsianum. New Phytol. 195, 189–202 (2012). 22497207 10.1111/j.1469-8137.2012.04129.x

[b35] Niehl A. *et al.* Control of Tobacco mosaic virus Movement Protein Fate by CELL-DIVISION-CYCLE Protein48. Plant Physiol. 160, 2093–2108 (2012). 23027663 10.1104/pp.112.207399 PMC3510134

[b36] Cheng Z. *et al.* Pathogen-secreted proteases activate a novel plant immune pathway. Nature 521, 213–216 (2015). 25731164 10.1038/nature14243 PMC4433409

[b37] Chen J.-G. *et al.* RACK1 mediates multiple hormone responsiveness and developmental processes in Arabidopsis. J. Exp. Bot. 57, 2697–2708 (2006). 16829549 10.1093/jxb/erl035

[b38] Jarsch I. K. & Ott T. Perspectives on Remorin Proteins, Membrane Rafts, and Their Role During Plant–Microbe Interactions. Mol. Plant. Microbe Interact. 24, 7–12 (2010). 21138374 10.1094/MPMI-07-10-0166

[b39] McKinney E. C., Kandasamy M. K. & Meagher R. B. Small changes in the regulation of one Arabidopsis profilin isovariant, PRF1, alter seedling development. Plant Cell 13, 1179–1191 (2001). 11340190 10.1105/tpc.13.5.1179 PMC135555

[b40] Abu-Abied M. *et al.* Identification of plant cytoskeleton-interacting proteins by screening for actin stress fiber association in mammalian fibroblasts. Plant J. 48, 367–379 (2006). 17010111 10.1111/j.1365-313X.2006.02883.x

[b41] Konopka-Postupolska D., Clark G. & Hofmann A. Structure, function and membrane interactions of plant annexins: An update. Plant Sci. 181, 230–241 (2011). 21763533 10.1016/j.plantsci.2011.05.013

[b42] Zhang Q. *et al.* Phosphatidic acid regulates microtubule organization by interacting with MAP65-1 in response to salt stress in Arabidopsis. Plant Cell 24, 4555–4576 (2012). 23150630 10.1105/tpc.112.104182 PMC3531852

[b43] Yu L. *et al.* Phosphatidic acid mediates salt stress response by regulation of MPK6 in *Arabidopsis thaliana*. New Phytol. 188, 762–773 (2010). 20796215 10.1111/j.1469-8137.2010.03422.x

[b44] Rancour D. M., Dickey C. E., Park S. & Bednarek S. Y. Characterization of AtCDC48. Evidence for Multiple Membrane Fusion Mechanisms at the Plane of Cell Division in Plants. Plant Physiol. 130, 1241–1253 (2002). 12427991 10.1104/pp.011742 PMC166645

[b45] Ishiguro S. *et al.* SHEPHERD is the Arabidopsis GRP94 responsible for the formation of functional CLAVATA proteins. EMBO J. 21, 898–908 (2002). 11867518 10.1093/emboj/21.5.898 PMC125899

[b46] Goeres D. C. *et al.* Components of the Arabidopsis mRNA Decapping Complex Are Required for Early Seedling Development. Plant Cell Online 19, 1549–1564 (2007). 10.1105/tpc.106.047621 PMC1913740 17513503

[b47] Frei dit Frey N. *et al.* Functional analysis of Arabidopsis immune-related MAPKs uncovers a role for MPK3 as negative regulator of inducible defences. Genome Biol. 15, R87 (2014). 24980080 10.1186/gb-2014-15-6-r87 PMC4197828

[b48] Park S., Rancour D. M. & Bednarek S. Y. In planta analysis of the cell cycle-dependent localization of AtCDC48A and its critical roles in cell division, expansion, and differentiation. Plant Physiol. 148, 246–258 (2008). 18660433 10.1104/pp.108.121897 PMC2528134

[b49] Smékalová V. *et al.* Involvement of YODA and mitogen activated protein kinase 6 in Arabidopsis post-embryogenic root development through auxin up-regulation and cell division plane orientation. New Phytol. 203, 1175–1193 (2014). 24923680 10.1111/nph.12880 PMC4414326

[b50] Hoehenwarter W. *et al.* Identification of novel *in vivo* MAP kinase substrates in *Arabidopsis thaliana* through use of tandem metal oxide affinity chromatography. Mol. Cell. Proteomics MCP 12, 369–380 (2013). 23172892 10.1074/mcp.M112.020560 PMC3567860

[b51] Popescu S. C. *et al.* MAPK target networks in *Arabidopsis thaliana* revealed using functional protein microarrays. Genes Dev. 23, 80–92 (2009). 19095804 10.1101/gad.1740009 PMC2632172

[b52] Chang R., Jang C. J. H., Branco-Price C., Nghiem P. & Bailey-Serres J. Transient MPK6 activation in response to oxygen deprivation and reoxygenation is mediated by mitochondria and aids seedling survival in Arabidopsis. Plant Mol. Biol. 78, 109–122 (2012). 22086331 10.1007/s11103-011-9850-5

[b53] Yoo S.-D., Cho Y.-H., Tena G., Xiong Y. & Sheen J. Dual control of nuclear EIN3 by bifurcate MAPK cascades in C2H4 signalling. Nature 451, 789–795 (2008). 18273012 10.1038/nature06543 PMC3488589

[b54] Lassowskat I., Böttcher C., Eschen-Lippold L., Scheel D. & Lee J. Sustained mitogen-activated protein kinase activation reprograms defense metabolism and phosphoprotein profile in *Arabidopsis thaliana. Front*. Plant Sci. 5, 554 (2014). 10.3389/fpls.2014.00554 PMC4202796 25368622

[b55] Beckers G. J. M. *et al.* Mitogen-Activated Protein Kinases 3 and 6 Are Required for Full Priming of Stress Responses in *Arabidopsis thaliana*. Plant Cell 21, 944–953 (2009). 19318610 10.1105/tpc.108.062158 PMC2671697

[b56] Yamada K., Nagano A. J., Nishina M., Hara-Nishimura I. & Nishimura M. Identification of Two Novel Endoplasmic Reticulum Body-Specific Integral Membrane Proteins. Plant Physiol. 161, 108–120 (2013). 23166355 10.1104/pp.112.207654 PMC3532245

[b57] Matsushima R., Kondo M., Nishimura M. & Hara-Nishimura I. A novel ER-derived compartment, the ER body, selectively accumulates a beta-glucosidase with an ER-retention signal in Arabidopsis. Plant J. Cell Mol. Biol. 33, 493–502 (2003). 10.1046/j.1365-313x.2003.01636.x 12581307

[b58] Berriri S. *et al.* Constitutively active mitogen-activated protein kinase versions reveal functions of Arabidopsis MPK4 in pathogen defense signaling. Plant Cell 24, 4281–4293 (2012). 23115249 10.1105/tpc.112.101253 PMC3517250

[b59] Xing Y. *et al.* MKK5 regulates high light-induced gene expression of Cu/Zn superoxide dismutase 1 and 2 in Arabidopsis. Plant Cell Physiol. 54, 1217–1227 (2013). 23677921 10.1093/pcp/pct072

[b60] Xing Y., Chen W., Jia W. & Zhang J. Mitogen-activated protein kinase kinase 5 (MKK5)-mediated signalling cascade regulates expression of iron superoxide dismutase gene in Arabidopsis under salinity stress. J. Exp. Bot. 66, 5971–5981 (2015). 26136265 10.1093/jxb/erv305 PMC4566985

[b61] Xing Y., Jia W. & Zhang J. AtMKK1 mediates ABA-induced CAT1 expression and H_2_O_2_ production via AtMPK6-coupled signaling in Arabidopsis. Plant J. 54, 440–451 (2008). 18248592 10.1111/j.1365-313X.2008.03433.x

[b62] Takáč T. *et al.* Proteomic and Biochemical Analyses Show a Functional Network of Proteins Involved in Antioxidant Defense of the Arabidopsis *anp2anp3* Double Mutant. J. Proteome Res. 13, 5347–5361 (2014). 25325904 10.1021/pr500588c PMC4423761

[b63] Baluska F. *et al.* Root hair formation: F-actin-dependent tip growth is initiated by local assembly of profilin-supported F-actin meshworks accumulated within expansin-enriched bulges. Dev. Biol. 227, 618–632 (2000). 11071779 10.1006/dbio.2000.9908

[b64] Li J., Blanchoin L. & Staiger C. J. Signaling to actin stochastic dynamics. Annu. Rev. Plant Biol. 66, 415–440 (2015). 25423079 10.1146/annurev-arplant-050213-040327

[b65] Murashige T. & Skoog F. A Revised Medium for Rapid Growth and Bio Assays with Tobacco Tissue Cultures. Physiol. Plant. 15, 473–497 (1962).

[b66] López-Bucio J. S. *et al.* *Arabidopsis thaliana* mitogen-activated protein kinase 6 is involved in seed formation and modulation of primary and lateral root development. J. Exp. Bot. 65, 169–183 (2014). 24218326 10.1093/jxb/ert368 PMC3883294

[b67] Takáč T., Pechan T., Šamajová O. & Šamaj J. Integrative chemical proteomics and cell biology methods to study endocytosis and vesicular trafficking in Arabidopsis. Methods Mol. Biol. Clifton NJ 1209, 265–283 (2014). 10.1007/978-1-4939-1420-3_20 25117290

[b68] Dinkel H. *et al.* ELM 2016-data update and new functionality of the eukaryotic linear motif resource. Nucleic Acids Res. 44, D294–300 (2016). 26615199 10.1093/nar/gkv1291 PMC4702912

[b69] Xue Y. *et al.* GPS: a comprehensive www server for phosphorylation sites prediction. Nucleic Acids Res. 33, W184–187 (2005). 15980451 10.1093/nar/gki393 PMC1160154

[b70] Bradford M. M. A rapid and sensitive method for the quantitation of microgram quantities of protein utilizing the principle of protein-dye binding. Anal. Biochem. 72, 248–254 (1976). 942051 10.1016/0003-2697(76)90527-3

[b71] Aebi H. Catalase *in vitro*. Methods Enzymol. 105, 121–126 (1984). 6727660 10.1016/s0076-6879(84)05016-3

[b72] Liu C. & Mehdy M. C. A nonclassical arabinogalactan protein gene highly expressed in vascular tissues, AGP31, is transcriptionally repressed by methyl jasmonic acid in Arabidopsis. Plant Physiol. 145, 863–874 (2007). 17885091 10.1104/pp.107.102657 PMC2048811

[b73] Hooks M. A. *et al.* Selective induction and subcellular distribution of ACONITASE 3 reveal the importance of cytosolic citrate metabolism during lipid mobilization in *Arabidopsis*. Biochem. J. 463, 309–317 (2014). 25061985 10.1042/BJ20140430

[b74] Taylor N. L., Heazlewood J. L., Day D. A. & Millar A. H. Lipoic acid-dependent oxidative catabolism of alpha-keto acids in mitochondria provides evidence for branched-chain amino acid catabolism in Arabidopsis. Plant Physiol. 134, 838–848 (2004). 14764908 10.1104/pp.103.035675 PMC344558

[b75] Xu J., Yang J.-Y., Niu Q.-W. & Chua N.-H. Arabidopsis DCP2, DCP1, and VARICOSE Form a Decapping Complex Required for Postembryonic Development. Plant Cell 18, 3386–3398 (2006). 17158604 10.1105/tpc.106.047605 PMC1785416

[b76] Winter G., Todd C. D., Trovato M., Forlani G. & Funck D. Physiological implications of arginine metabolism in plants. Front Plant Sci 6, 534 (2015). 26284079 10.3389/fpls.2015.00534 PMC4520006

[b77] Sung D. Y., Vierling E. & Guy C. L. Comprehensive Expression Profile Analysis of the Arabidopsis Hsp70 Gene Family. Plant Physiol. 126, 789–800 (2001). 11402207 10.1104/pp.126.2.789 PMC111169

[b78] Guo Y., Xiong L., Ishitani M. & Zhu J.-K. An Arabidopsis mutation in translation elongation factor 2 causes superinduction of CBF/DREB1 transcription factor genes but blocks the induction of their downstream targets under low temperatures. Proc. Natl. Acad. Sci. USA 99, 7786–7791 (2002). 12032361 10.1073/pnas.112040099 PMC124352

[b79] Gookin T. E. & Assmann S. M. Significant reduction of BiFC non-specific assembly facilitates in planta assessment of heterotrimeric G-protein interactors. Plant J. 80, 553–567 (2014). 25187041 10.1111/tpj.12639 PMC4260091

[b80] Park S., Rancour D. M. & Bednarek S. Y. In planta analysis of the cell cycle-dependent localization of AtCDC48A and its critical roles in cell division, expansion, and differentiation. Plant Physiol. 148, 246–258 (2008). 18660433 10.1104/pp.108.121897 PMC2528134

[b81] Heeg C. *et al.* Analysis of the Arabidopsis O-acetylserine(thiol)lyase gene family demonstrates compartment-specific differences in the regulation of cysteine synthesis. Plant Cell 20, 168–185 (2008). 18223034 10.1105/tpc.107.056747 PMC2254930

[b82] Mayfield J. D., Paul A.-L. & Ferl R. J. The 14-3-3 proteins of Arabidopsis regulate root growth and chloroplast development as components of the photosensory system. J. Exp. Bot. 63, 3061–3070 (2012). 22378945 10.1093/jxb/ers022 PMC3350920

[b83] Deyholos M. K. *et al.* VARICOSE, a WD-domain protein, is required for leaf blade. Development 130, 6577–6588 (2003). 14660546 10.1242/dev.00909

[b84] Jin H., Song Z. & Nikolau B. J. Reverse genetic characterization of two paralogous acetoacetyl CoA thiolase genes in Arabidopsis reveals their importance in plant growth and development. Plant J. 70, 1015–1032 (2012). 22332816 10.1111/j.1365-313X.2012.04942.x

[b85] Suarez M. F. *et al.* Metacaspase-dependent programmed cell death is essential for plant embryogenesis. Curr. Biol. CB 14, R339–340 (2004). 15120084 10.1016/j.cub.2004.04.019

[b86] Guo D. *et al.* Cis-cinnamic acid-enhanced 1 gene plays a role in regulation of Arabidopsis bolting. Plant Mol. Biol. 75, 481–495 (2011). 21298397 10.1007/s11103-011-9746-4

[b87] Suzuki K. *et al.* Plastid chaperonin proteins Cpn60α and Cpn60β are required for plastid division in *Arabidopsis thaliana*. BMC Plant Biol. 9, 38 (2009). 19344532 10.1186/1471-2229-9-38 PMC2670834

[b88] Chen M. & Thelen J. J. The plastid isoform of triose phosphate isomerase is required for the postgerminative transition from heterotrophic to autotrophic growth in Arabidopsis. Plant Cell 22, 77–90 (2010). 20097871 10.1105/tpc.109.071837 PMC2828694

[b89] Ma X., Song L., Yang Y. & Liu D. A gain-of-function mutation in the ROC1 gene alters plant architecture in Arabidopsis. New Phytol. 197, 751–762 (2013). 23206262 10.1111/nph.12056

[b90] Strompen G. *et al.* Arabidopsis vacuolar H-ATPase subunit E isoform 1 is required for Golgi organization and vacuole function in embryogenesis. Plant J. Cell Mol. Biol. 41, 125–132 (2005). 10.1111/j.1365-313X.2004.02283.x 15610355

[b91] Uváčková Ľ., Takáč T., Boehm N., Obert B. & Šamaj J. Proteomic and biochemical analysis of maize anthers after cold pretreatment and induction of androgenesis reveals an important role of anti-oxidative enzymes. J. Proteomics 75, 1886–1894 (2012). 22252011 10.1016/j.jprot.2011.12.033

[b92] Kim S. Y. & Nam K. H. Physiological roles of ERD10 in abiotic stresses and seed germination of Arabidopsis. Plant Cell Rep. 29, 203–209 (2010). 20054552 10.1007/s00299-009-0813-0

[b93] Pereira L. A. R. *et al.* Methyl recycling activities are co-ordinately regulated during plant development. J. Exp. Bot. 58, 1083–1098 (2007). 17272833 10.1093/jxb/erl275

[b94] Yang M., Hu Y., Lodhi M., McCombie W. R. & Ma H. The Arabidopsis SKP1-LIKE1 gene is essential for male meiosis and may control homologue separation. Proc. Natl. Acad. Sci. USA 96, 11416–11421 (1999). 10500191 10.1073/pnas.96.20.11416 PMC18048

[b95] Yang X., Timofejeva L., Ma H. & Makaroff C. A. The Arabidopsis SKP1 homolog ASK1 controls meiotic chromosome remodeling and release of chromatin from the nuclear membrane and nucleolus. J. Cell Sci. 119, 3754–3763 (2006). 16940350 10.1242/jcs.03155

[b96] Porat R., Lu P. & O’Neill S. D. Arabidopsis SKP1, a homologue of a cell cycle regulator gene, is predominantly expressed in meristematic cells. Planta 204, 345–351 (1998). 9530878 10.1007/s004250050265

[b97] Liu F. *et al.* The ASK1 and ASK2 genes are essential for Arabidopsis early development. Plant Cell 16, 5–20 (2004). 14688296 10.1105/tpc.017772 PMC301391

[b98] Kim J. Y. *et al.* Functional characterization of a glycine-rich RNA-binding protein 2 in *Arabidopsis thaliana* under abiotic stress conditions. Plant J. 50, 439–451 (2007). 17376161 10.1111/j.1365-313X.2007.03057.x

[b99] Fusaro A. F. *et al.* AtGRP2, a cold-induced nucleo-cytoplasmic RNA-binding protein, has a role in flower and seed development. Planta 225, 1339–1351 (2007). 17123099 10.1007/s00425-006-0444-4

[b100] Linkies A. *et al.* Ethylene Interacts with Abscisic Acid to Regulate Endosperm Rupture during Germination: A Comparative Approach Using *Lepidium sativum* and *Arabidopsis thaliana*. Plant Cell 21, 3803–3822 (2009). 20023197 10.1105/tpc.109.070201 PMC2814513

[b101] Qin Y.-M. *et al.* Saturated very-long-chain fatty acids promote cotton fiber and Arabidopsis cell elongation by activating ethylene biosynthesis. Plant Cell 19, 3692–3704 (2007). 17993622 10.1105/tpc.107.054437 PMC2174872

[b102] Péret B. *et al.* Auxin regulates aquaporin function to facilitate lateral root emergence. Nat. Cell Biol. 14, 991–998 (2012). 22983115 10.1038/ncb2573

[b103] Shen L., Kang Y. G. G., Liu L. & Yu H. The J-domain protein J3 mediates the integration of flowering signals in Arabidopsis. Plant Cell 23, 499–514 (2011). 21343416 10.1105/tpc.111.083048 PMC3077791

[b104] Renault H. *et al.* γ-Aminobutyric acid transaminase deficiency impairs central carbon metabolism and leads to cell wall defects during salt stress in Arabidopsis roots. Plant Cell Environ. 36, 1009–1018 (2013). 23148892 10.1111/pce.12033

[b105] Kandasamy M. K., McKinney E. C. & Meagher R. B. A single vegetative actin isovariant overexpressed under the control of multiple regulatory sequences is sufficient for normal Arabidopsis development. Plant Cell 21, 701–718 (2009). 19304937 10.1105/tpc.108.061960 PMC2671709

[b106] Thitamadee S., Tuchihara K. & Hashimoto T. Microtubule basis for left-handed helical growth in Arabidopsis. Nature 417, 193–196 (2002). 12000963 10.1038/417193a

[b107] Matsumoto S. *et al.* Gravity-induced modifications to development in hypocotyls of Arabidopsis tubulin mutants. Plant Physiol. 152, 918–926 (2010). 20018592 10.1104/pp.109.147330 PMC2815900

[b108] Kandasamy M. K., Gilliland L. U., McKinney E. C. & Meagher R. B. One plant actin isovariant, ACT7, is induced by auxin and required for normal callus formation. Plant Cell 13, 1541–1554 (2001). 11449050 10.1105/TPC.010026 PMC139544

[b109] Gilliland L. U., Pawloski L. C., Kandasamy M. K. & Meagher R. B. Arabidopsis actin gene ACT7 plays an essential role in germination and root growth. Plant J. Cell Mol. Biol. 33, 319–328 (2003). 10.1046/j.1365-313x.2003.01626.x 12535345

[b110] Uraji M. *et al.* Cooperative function of PLDδ and PLDα1 in abscisic acid-induced stomatal closure in Arabidopsis. Plant Physiol. 159, 450–460 (2012). 22392280 10.1104/pp.112.195578 PMC3375977

[b111] Guo J. & Chen J.-G. RACK1 genes regulate plant development with unequal genetic redundancy in Arabidopsis. BMC Plant Biol. 8, 108 (2008). 18947417 10.1186/1471-2229-8-108 PMC2577656

[b112] Vellosillo T. *et al.* Oxylipins produced by the 9-lipoxygenase pathway in Arabidopsis regulate lateral root development and defense responses through a specific signaling cascade. Plant Cell 19, 831–846 (2007). 17369372 10.1105/tpc.106.046052 PMC1867370

[b113] Gallois J.-L. *et al.* The Arabidopsis proteasome RPT5 subunits are essential for gametophyte development and show accession-dependent redundancy. Plant Cell 21, 442–459 (2009). 19223514 10.1105/tpc.108.062372 PMC2660631

[b114] Ueda M. *et al.* Arabidopsis RPT2a Encoding the 26S Proteasome Subunit is Required for Various Aspects of Root Meristem Maintenance, and Regulates Gametogenesis Redundantly with its Homolog, RPT2b. Plant Cell Physiol. 52, 1628–1640 (2011). 21784786 10.1093/pcp/pcr093

[b115] Hossain Z. *et al.* The translation elongation factor eEF-1Bβ1 is involved in cell wall biosynthesis and plant development in *Arabidopsis thaliana*. PloS One 7, e30425 (2012). 22272350 10.1371/journal.pone.0030425 PMC3260303

