# Supplementary material for: Comparative proteomic study of Arabidopsis mutants mpk4 and mpk6
Source: Sci Rep. 2016 Jun 21;6:28306. doi: 10.1038/srep28306 (PMC4915016; doi:10.1038/srep28306)
Supplement: Supplementary Information [file srep28306-s1.pdf]

Supplementary information

Comparative proteomic study of Arabidopsis mutants *mpk4* and *mpk6*

Tomáš Takáč[1], Pavol Vadovič[1], Tibor Pechan[2], Ivan Luptovčiak[1], Olga Šamajová[1], Jozef Šamaj[1*]

[1]Centre of the Region Haná for Biotechnological and Agricultural Research, Faculty of Science, Palacký University, Šlechtitelů 27, 783 71 Olomouc, Czech Republic
[2] Institute for Genomics, Biocomputing & Biotechnology, Mississippi State University, MS 39762, USA

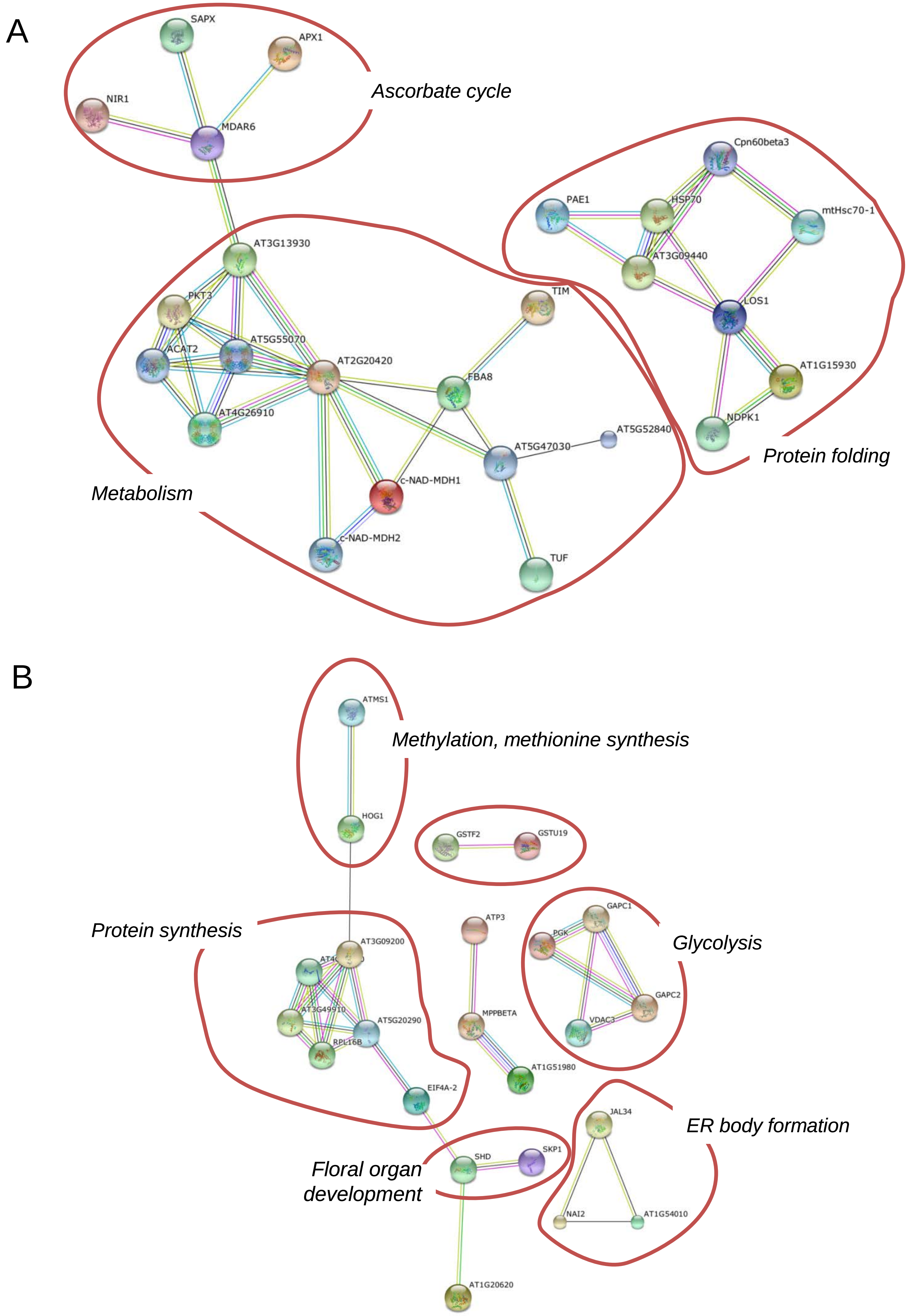

Figure S1 Interaction networks in *mpk4* mutant differential root proteome as predicted by STRING software for proteins with decreased (A) and increased (B) abundance in the mutant compared to wild type.

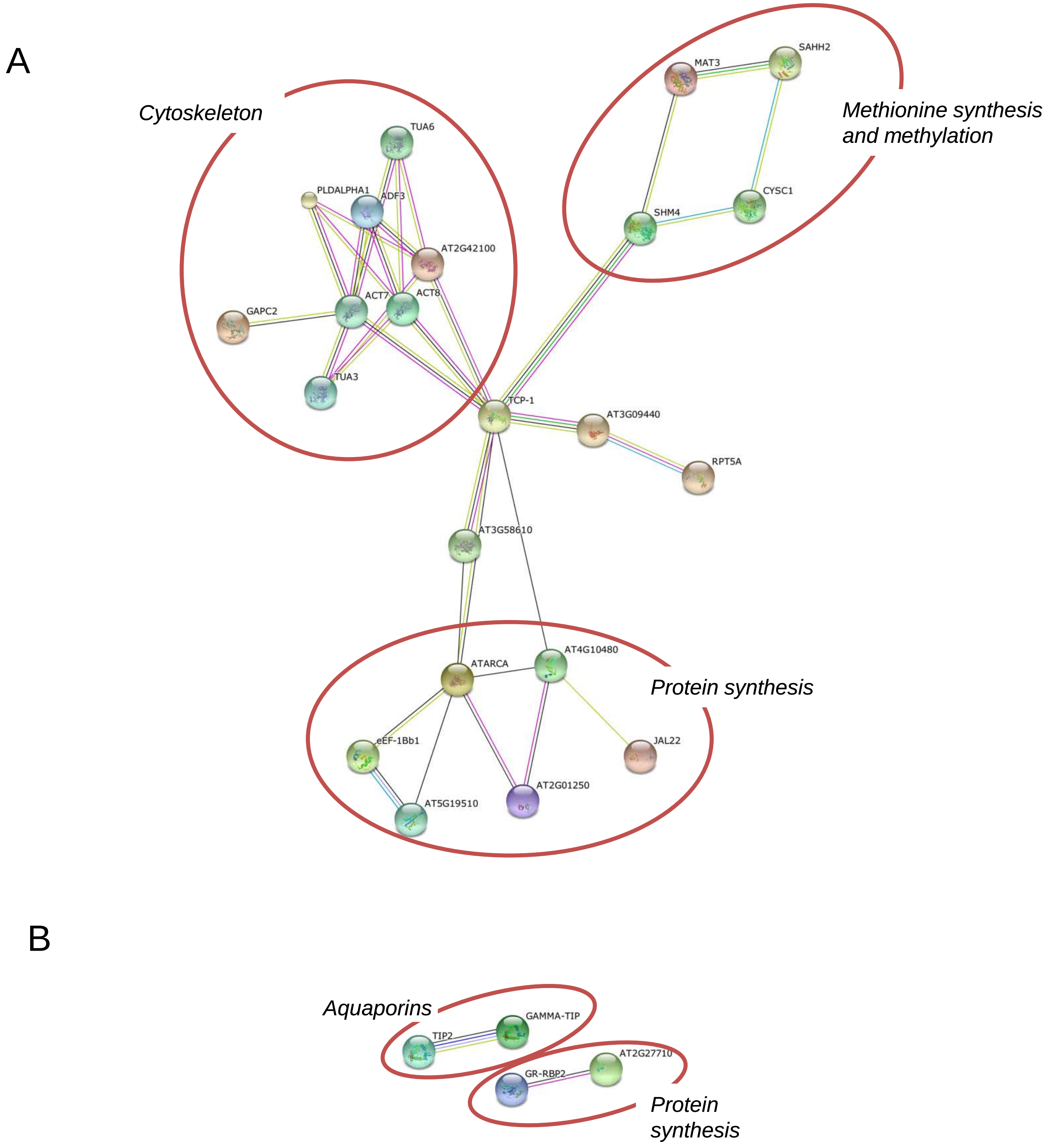

Figure S2 Interaction networks in *mpk6* mutant differential root proteome as predicted by STRING software for proteins with decreased (A) and increased (B) abundance in the mutant compared to wild type.

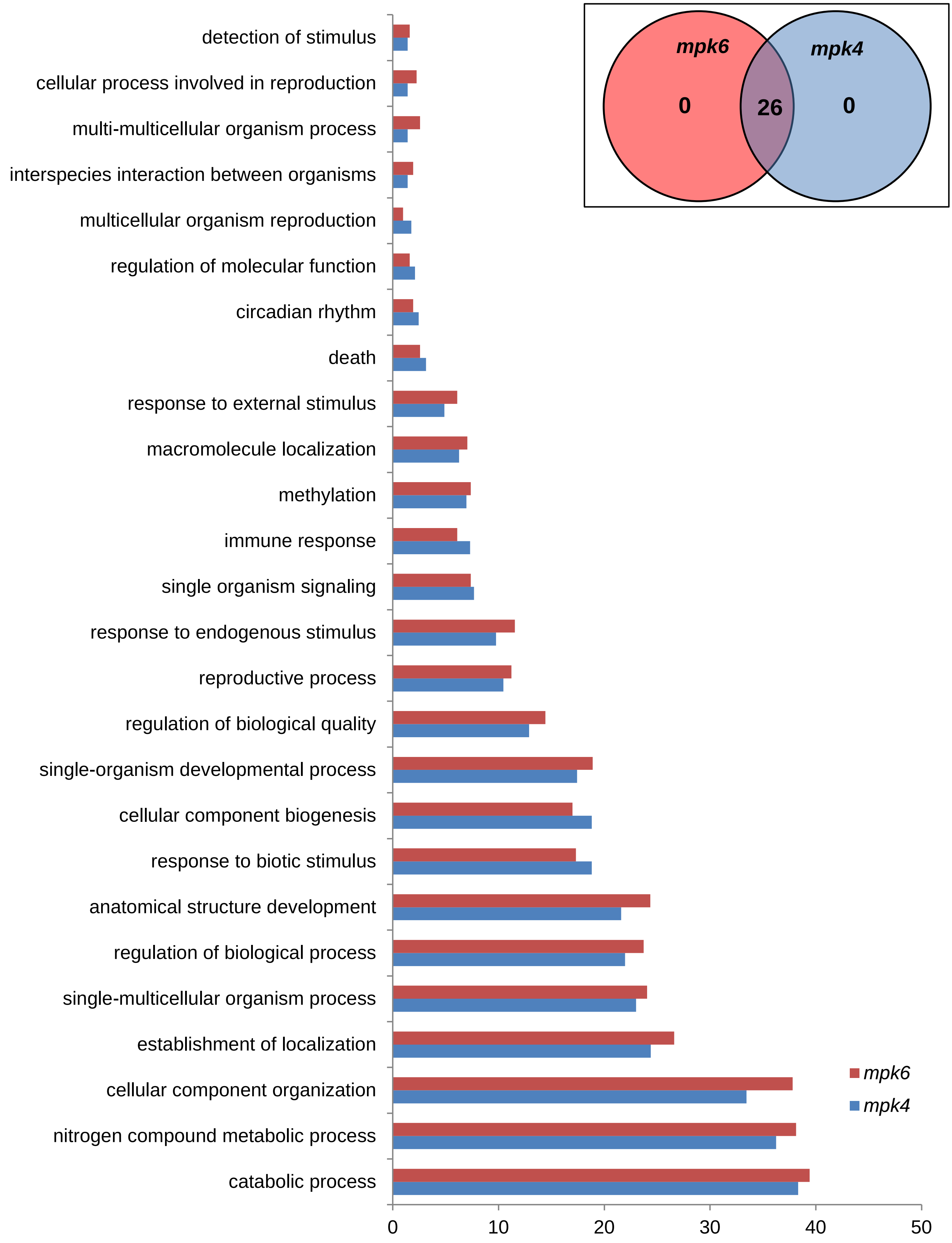

Figure S3. Comparison of gene onthology annotations (at 3rd level of onthology) of all proteins identified in *mpk4* and *mpk6* mutant roots according to biological process. Relative protein numbers of proteins per annotation are presented in the graph. Venn diagram shows the number of GO annotations assigned solely to *mpk6* (red field) and *mpk4* mutant root proteome (blue field) as well as the number of GO annotations present in both mutant proteomes (overlap).

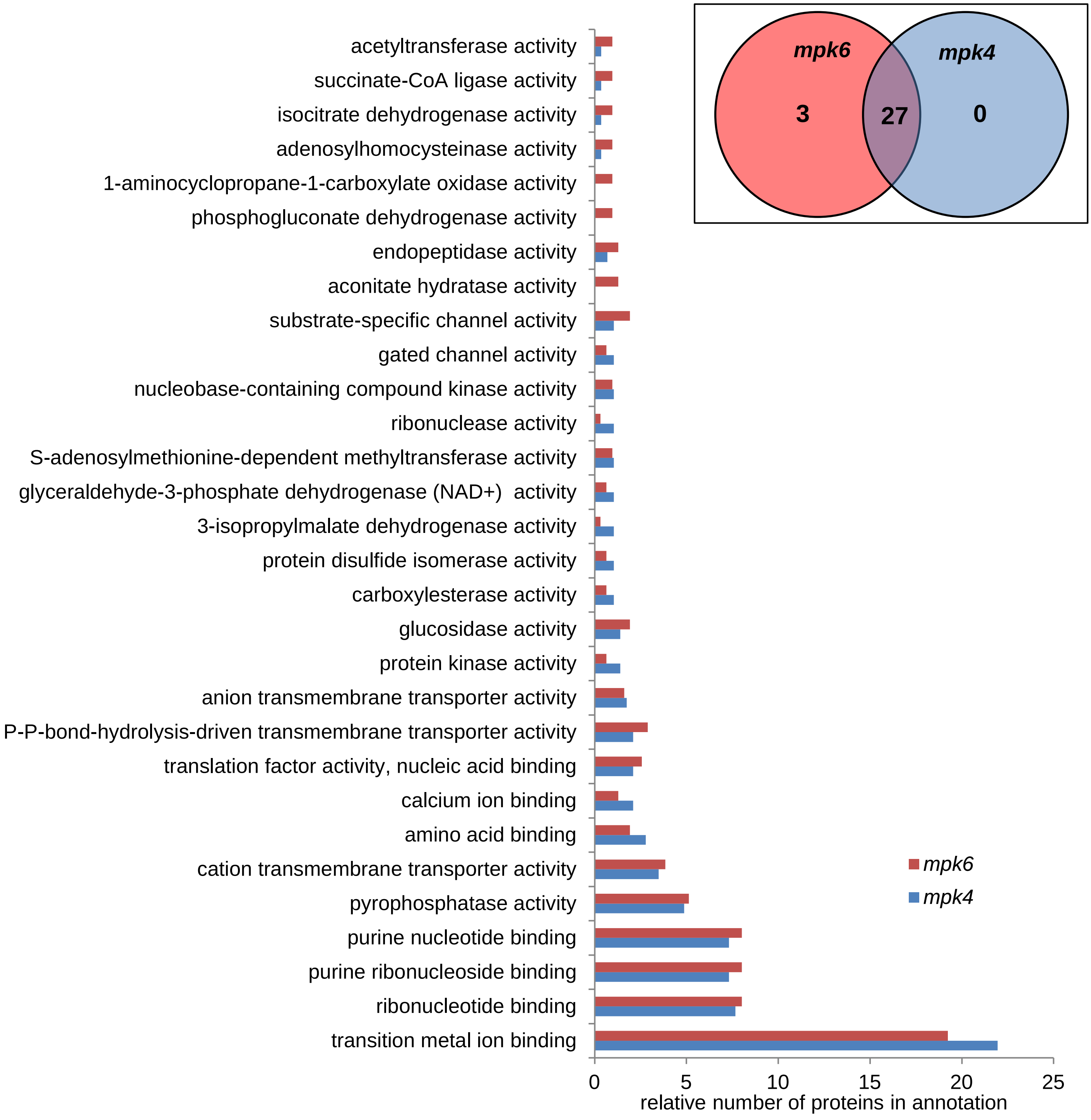

Figure S4. Comparison of gene onthology annotations (at 6th level of onthology) of all proteins identified in *mpk4* and *mpk6* mutant roots according to molecular function. Relative protein numbers of proteins per annotation are presented in the graph. Venn diagram shows the number of GO annotations assigned solely to *mpk6* (red field) and *mpk4* mutant root proteome (blue field) as well as the number of GO annotations present in both mutant proteomes (overlap).

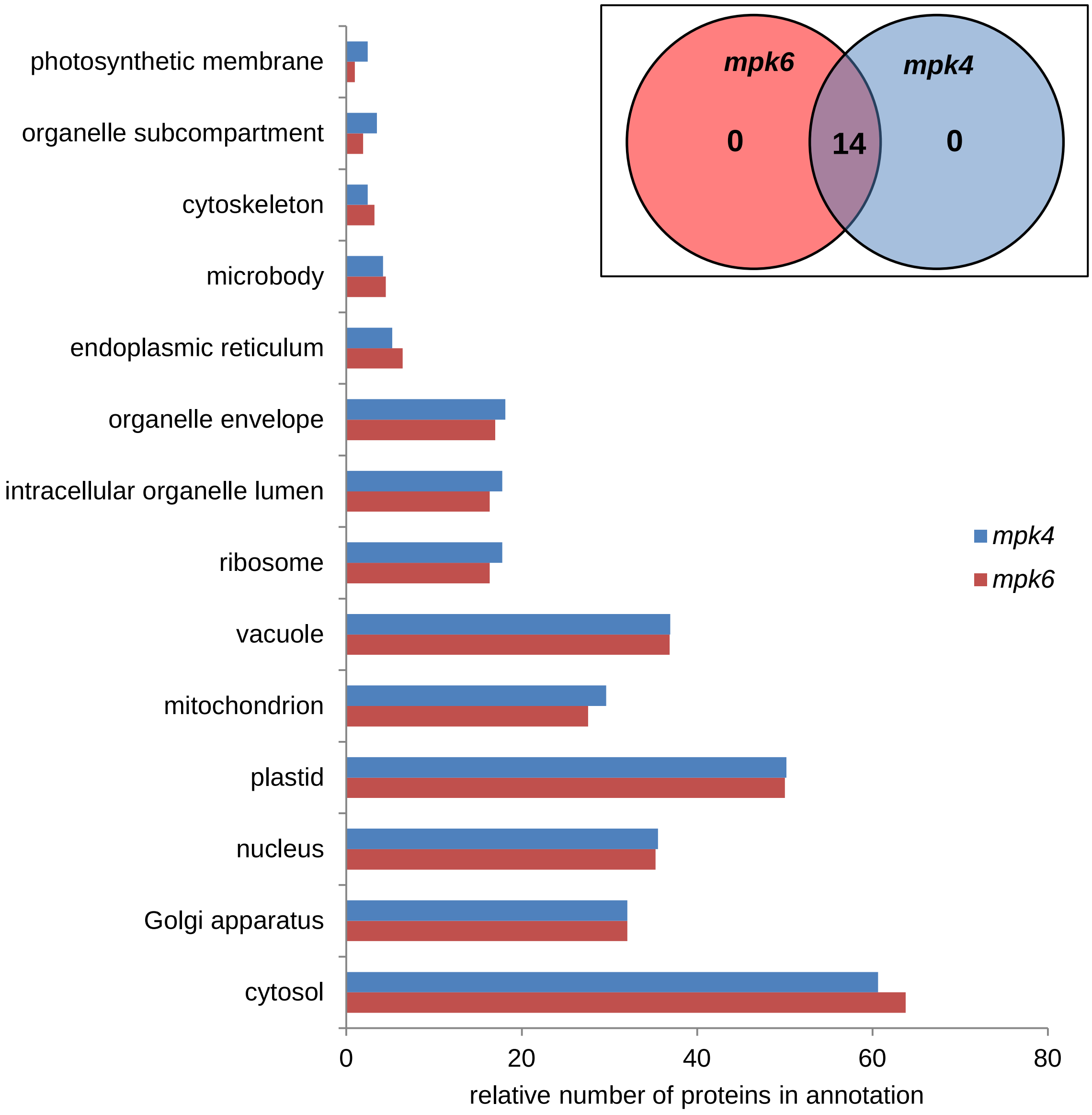

Figure S5. Comparison of gene onthology annotations (at 8th level of onthology) of all proteins identified in *mpk4* and *mpk6* mutant roots (in 3 out of 4 biological replicates) according to cellular component. Relative protein numbers of proteins per annotation are presented in the graph. Venn diagram shows the number of GO annotations assigned solely to *mpk6* (red field) and *mpk4* mutant root proteome (blue field) as well as the number of GO annotations present in both mutant proteomes (overlap).

Table S1. List of proteins with significantly changed abundance in roots of Arabidopsis *mpk4* mutant seedlings compared to wild type (Col-0). Abundance was calculated from 4 biological replicates. One way Anova analysis was carried out for statistical evaluation of significance.

Accession	Coverage	# Peptides	MW [kDa]	calc. pI	Score	Description	Intensity sum (average Col0)	Intensity sum (average *mpk4*)	*P* -value	fold
**DOWNREGULATED**										
**Stress related proteins**										
gi334185401	34.34	17	32.1	5.44	82.86	JA-responsive protein 1	44159838	28287813	0.023	0.64
gi18413214	29.59	9	18.8	8.27	61.76	nucleoside diphosphate kinase 1	37876742	23032132	0.045	0.61
gi15224648	30	6	11	4.88	43.39	membrane-associated progesterone binding protein 2	45080293	16934108	0.001	0.38
gi18379240	46.36	24	17.5	5.73	142.14	MLP-like protein 328	133320704	46183710	0.001	0.35
gi18379244	49.01	23	17.6	5.55	128.66	MLP-like protein 329	92297424	23811471	0.001	0.26
gi334186408	17.5	10	37.4	9.6	55.92	L-ascorbate peroxidase S	16727486	1908328	0.022	0.11
gi30696930	21.37	18	52.1	7.75	122.47	Monodehydroascorbate reductase	45865373	10864237	0.001	0.24
gi30686836	25.1	17	29.4	5.47	75.07	dehydrin ERD10	24023126	7804980	0.02	0.32
gi30690396	31.25	41	38.8	6.67	258.53	Peroxidase family protein	168956530	70979878	0.016	0.42
gi30690772	34.22	29	29.2	8.5	153.92	glutathione S-transferase phi 8	99466734	49415219	0.001	0.5
gi145323784	45.78	34	27.5	6.29	181.81	L-ascorbate peroxidase 1	78213410	18975297	0.041	0.24
gi15237716	13.42	5	33.8	6.1	30.87	TOMV RNA binding protein	12474685	2640969	0.001	0.21
gi15232058	13.4	6	34.9	8.95	25.84	peroxidase 27	4017186	-	-	unique in WT
gi18401423	11.93	1	12.2	5.68	14	heat stable protein 1	2733535	-	-	unique in WT
gi145334501	5.84	1	27	5.63	22.57	binding partner of acd11 1	4012129	-	-	unique in WT
**Protein synthesis and folding**										
gi334188446	16.92	18	63.3	5.87	104.79	TCP-1/cpn60 chaperonin family protein	44038838	10240942	0.007	0.23
gi15230534	23.54	34	71.1	5.25	208.69	heat shock protein 70-4	113395522	-	-	unique in WT
gi334185190	26.81	44	71.1	5.7	229.3	heat shock protein 70-3	144132985	82821161	0.008	0.57
gi240255880	53.49	11	9.3	4.48	61.9	heat shock factor binding protein	21432992	-	-	unique in WT
gi30691626	14.37	19	73	5.62	147.99	heat shock protein 70-1	37202366	11969206	0.015	0.32
gi15236211	43.02	19	18.4	8.69	168.3	rotamase cyclophilin 5	99205263	49647915	0.004	0.5
gi30684617	29.17	11	15.4	5.55	64.37	40S ribosomal protein S12-1	31597439	4316075	0.004	0.14
gi15234781	60.47	19	18.4	7.81	130.6	peptidyl-prolyl cis-trans isomerase CYP1	84563207	45953462	0.005	0.54
gi30696056	14	25	93.8	6.25	161.39	elongation factor EF-2	36188952	2767139	0.001	0.08
**Vesicular trafficking**										
gi15237054	30.87	19	26	6.4	176.65	V-type proton ATPase subunit E1	579193317	6277232	0.015	0.11
**Cytoskeletal proteins**										
gi15233538	43.51	15	14	5.2	73	profilin 2	49367881	12542005	0.004	0.25
gi15224838	43.51	11	14.3	4.82	72.27	profilin 1	28135567	10004823	0.020	0.36
**Metabolism**										
gi79558700	17.16	6	19	6.79	32.4	rhodanese-like domain-containing protein	8990279	-	-	unique in WT

gi334186360	25.58	4	14	5.26	22.15	peptide methionine sulfoxide reductase B5	7918550	-	-	unique in WT
gi30695409	4.27	1	40.9	6.5	23.45	acetoacetyl-CoA thiolase 2	4097356	-	-	unique in WT
gi18423437	8.88	1	19.2	4.77	3.67	probable NADH dehydrogenase [ubiquinone] 1 alpha subcomplex subunit 5	14759169	3307172	0.018	0.22
gi15225353	8.31	7	45.3	6.71	88.83	Succinyl-CoA ligase [GDP-forming] subunit beta	24356937	8969895	0.021	0.37
gi15219721	38.86	32	35.5	6.55	225.34	malate dehydrogenase	146565660	60763875	0.016	0.41
gi238479568	9.82	3	18.5	4.78	16.32	thiocyanate methyltransferase 1	6653891	2836376	0.017	0.43
gi145329204	22.88	13	32.3	7.49	75.34	triosephosphate isomerase	26467098	11954894	0.041	0.45
gi15225798	24.68	14	48.5	8.34	87.2	3-ketoacyl-CoA thiolase 2	22599512	10566258	0.028	0.47
gi15219886	15.18	10	21.2	4.34	45.56	cytochrome C oxidase 6B	27322231	14556289	0.009	0.53
gi15236568	14.57	8	17.5	6.34	43.47	polyketide cyclase/dehydrase and lipid transport superfamily protein	38732855	10718416	0.018	0.28
gi79313339	20.76	5	25.5	5.2	26.99	alpha/beta-Hydrolases superfamily protein	8443513	2694350	0.024	0.32
gi186502696	24.42	27	54.5	5.92	129.1	aminopeptidase family protein	39638087	24594163	0.035	0.62
gi30687411	16.99	5	39.7	7.53	44.11	Dihydrolipoamide succinyltransferase	9641136	-	-	unique in WT
gi18400212	4.45	2	58.4	7.65	31.36	dihydrolipoamide acetyltransferase, long form protein	11109967	-	-	unique in WT
gi15240454	3.66	4	50.1	9.14	25.37	dihydrolipoyllysine-residue succinyltransferase component of 2-oxoglutarate dehydrogenase complex 1	728437	-	-	unique in WT
gi15239843	24.7	21	35.7	6.79	146.81	malate dehydrogenase	94027894	-	-	unique in WT
gi15237998	7.88	4	21.5	6.7	22.47	ATP synthase subunit delta'	6292429	-	-	unique in WT
gi15237679	14.29	5	12	6.4	32.07	peptidyl-prolyl isomerase FKBP12	7765998	-	-	unique in WT
gi15233349	6.46	13	98.1	6.4	78.55	aconitate hydratase 1	7324322	-	-	unique in WT
gi15226573	4.78	7	65.5	6.28	43.64	Ferredoxin--nitrite reductase	3798383	-	-	unique in WT
gi15231715	50.56	43	38.5	6.46	272.67	fructose-bisphosphate aldolase	166335365.1	117852344.5	0.025	0.71
						**Signaling**				
gi18417863	13.6	5	30.2	4.81	36.66	14-3-3-like protein GF14 upsilon	3761124	-	-	unique in WT
						**Proteolysis**				
gi22331076	6.44	2	81.8	6.77	30.68	Subtilase family protein	3949056	-	-	unique in WT
gi15220874	27.55	13	22.1	8.78	63.97	kunitz type trypsin and protease inhibitor domain-containing protein	13269998	-	-	unique in WT
gi145325425	20.25	6	25.9	4.75	59.01	proteasome subunit alpha type-5-A	6712319	2157322	0.023	0.32
gi15219345	16.27	9	45.5	4.82	59.27	metacaspase 4	14305198	-	-	unique in WT
						**Unknown function**				
gi240254562	3	6	148.5	4.75	81.64	uncharacterized protein	12034279	-	-	unique in WT
gi18402503	33.54	4	16.9	5.3	33.78	uncharacterized protein	4479077	-	-	unique in WT
						**Cell wall composition**				
gi145324054	6.3	5	33.9	10.7	42.42	arabinogalactan protein 31	5011771	-	-	unique in WT
						**Translation**				
gi30682607	1.6	3	141.9	6.6	25.44	mRNA decapping complex VCS	122872	-	-	unique in WT
						**Scaffolding protein**				
gi15225899	13.68	3	21	8.54	18.85	Remorin family protein	7158948	-	-	unique in WT

					**UPREGULATED**					
					**Metabolism**					
gi15227257	22.46	10	35.4	8.9	81.77	ATP synthase subunit gamma	3880740	18269069	0.002	4.71
gi15218090	16.1	8	54.4	6.33	80.45	putative mitochondrial-processing peptidase subunit alpha-1	3945991	51746913	0.038	2.31
gi15240765	16.67	5	29.6	8.85	64.56	voltage dependent anion channel 2	4835579	11676030	0.005	2.41
gi79587736	18.2	10	31.9	5.27	55.37	glyoxalase I homolog	5433689	38446250	0.001	7.08
gi79327847	31.7	26	41.9	6	125.33	isopropylmalate dehydrogenase 1	21088922	50750377	0.007	2.41
gi15229231	50	55	36.9	7.12	308.86	glyceraldehyde-3-phosphate dehydrogenase C subunit 1	141154062	370726048	0.004	2.63
gi145334507	23.1	57	84.3	6.51	317.79	5-methyltetrahydropteroyltriglutamate--homocysteine methyltransferase	43824674	120126136	0.025	2.74
gi15235213	24.71	13	29.1	5.29	89.73	putative caffeoyl-CoA O-methyltransferase	16286350	37972542	0.027	2.33
gi30699430	34.66	27	42.1	5.68	203.37	phosphoglycerate kinase	83143041	131826906	0.016	1.59
					**Nucleic acid binding**					
gi79313257	25.2	24	80.7	4.7	221.13	DNA topoisomerase-like protein	44648919	74541493	0.001	1.67
gi15239697	24.62	8	13.6	10.15	39.65	histone H2A	8672749	19871188	0.021	2.29
					**Stress related**					
gi15220216	34.7	20	36.2	5.38	159.85	annexin 1	18417212	81194961	0.013	4.41
gi240255920	34.3	23	37.7	6.84	144.83	putative beta-1,3-endoglucanase	14524276	86520500	0.004	5.96
gi18414298	22.7	13	47.5	5.31	107.75	monodehydroascorbate reductase (NADH)	9202420	56147858	0.046	6.1
gi15235401	38.21	23	24.1	6.35	146.9	glutathione S-transferase F2	41864796	118071075	0.021	2.82
gi18411929	12.79	2	25.6	6.4	65.94	glutathione S-transferase TAU 19	4073923	12470006	0.013	3.06
gi79607896	31.91	60	72.4	5.5	368.81	jacalin-like lectin domain-containing protein	102024107	373005179	0.001	3.66
					**Protein synthesis, processing and folding**					
gi15236981	17.91	2	15.5	10.4	28.17	60S ribosomal protein L14-2	4460163	32625377	0.001	7.31
gi30694819	13.95	4	19.8	9.92	34.55	60S ribosomal protein L11-2	2227646	21059651	0.005	9.45
gi145332383	13.43	6	45.7	6.28	42.16	translational initiation factor 4A-1	5401363	15502069	0.043	2.87
gi18410311	12.56	6	23.8	7.18	59.49	kunitz trypsin inhibitor 1	3790257	13873594	0.001	3.66
gi15232603	25	14	34.1	5.7	104.3	60S acidic ribosomal protein P0-2	3050007	14057122	0.044	4.61
gi15233740	15.67	20	94.1	5.3	141.95	HSP90-like protein GRP94	9673127	48278582	0.000	4.99
gi15233268	34	13	27.5	7.11	64.98	20S proteasome alpha-3 subunit	2774847	15100795	0.012	5.44
					**Lipid metabolism**					
gi18404748	28.76	26	43.1	8.7	164.62	GDSL esterase/lipase	18672028	102368760	0.005	5.48
gi15232845	25.42	28	59.1	6.79	183.49	probable mitochondrial-processing peptidase subunit beta	3945991	51746913	0.001	13.11
					**AminoAcid metabolism**					
gi186513287	3.78	3	49	7.72	18.56	argininosuccinate synthase	1422378	5166958	0.041	3.63
gi145333043	14.77	10	35.5	5.16	61	adenosylhomocysteinase	892125	22523603	0.015	25.25
					**Unknown**					
gi15229809	5.88	8	21.6	9.41	26.53	uncharacterized protein	10940894	28197085	0.047	2.58
gi22328599	18.39	3	9.5	4.97	24.38	RNA recognition motif-containing protein		4138353		unique in *mpk4*
gi186478702	3.71	3	43.9	7.61	17.48	catalase 3	-	9288542	-	unique in *mpk4*

gi18421006	30.77	19	39.8	9.22	106.73	TRAF-like family protein	-	77036438	-	unique in *mpk4*
gi18410982	9.38	4	17.8	4.54	18.16	S-phase kinase-associated protein 1	-	2756551	-	unique in *mpk4*
gi18406229	13.89	15	45	5.82	76.81	TRAF-like protein	-	2756551	-	unique in *mpk4*
gi15241316	33.78	17	25	10.32	87.57	40S ribosomal protein S8-1	-	55838806	-	unique in *mpk4*
gi15229631	15.7	3	16.9	10.78	36.15	60S ribosomal protein L26-1	-	16600695	-	unique in *mpk4*
gi15226503	6.61	3	26.9	7.2	16.1	short-chain dehydrogenase reductase 3a	-	8057351	-	unique in *mpk4*

Table S2. List of proteins with significantly changed abundance in roots of Arabidopsis *mpk6* mutant seedlings compared to wild type (Col-0). Abundance was calculated from 4 biological replicates. One way Anova analysis was carried out for statistical evaluation of significance.

Accession	Coverage	# Peptides	MW [kDa]	calc. pI	Score	Description	Intensity sum (average Col0)	Intensity sum (average *mpk6*)	Fold change	*P* value
**DOWNREGULATED**										
**Stress related proteins**										
gi15228041	35.3	4	41.8	6.51	47.67	aquaporin TIP1-1	12761819	672496	0.05	1.02E-05
gi15231569	32.38	7	41.8	5.6	42.67	aquaporin TIP1-2	10500652	813310	0.08	0.005
gi18414298	29.49	8	47.5	4.97	56.54	monodehydroascorbate reductase (NADH)	55562447	1493058	0.03	0.01
**Cytoskeletal proteins**										
gi15233538	41.66	7	14	5.2	46.79	profilin 2	94136176	19684596	0.21	0.049
**Metabolism**										
gi15220770	41.79	1	36.2	5.12	44.348	1-aminocyclopropane-1-carboxylate oxidase 2	4871669	462263	0.09	0.032
gi15221156	32.66	5	61.4	6.1	49.41	pyrophosphate--fructose-6-phosphate 1-phosphotransferase	31023724	2581092	0.08	0.01
gi15240628	41.86	2	41.72	9.86	40.65	delta subunit of Mt ATP synthase	39349332	8045791	0.2	0.03
gi15241592	30.25	10	36.1	8.39	70.64	Succinyl-CoA ligase [GDP-forming] subunit alpha-1	85150756	18785954	0.22	0.01
gi18403295	41.8	8	55.2	8.43	61.33	gamma-aminobutyrate transaminase POP2	32812295	4583895	0.14	0.015
**Protein synthesis and folding**										
gi30691988	41.73	1	37.7	8.18	41426	chaperone protein dnaJ 3	31023724	2581092	0.14	0.022
gi15226231	48.7	15	41.7	4.61	86.42	60S acidic ribosomal protein P2-1	142985326	58068438	0.41	0.021
gi15240317	41.95	6	60.3	5.47	56.61	TCP-1/cpn60 chaperonin family protein	7454995	1014447	0.14	0.03
gi18394184	27.55	5	41.86	7.69	45.84	chaperonin 10	150262741	13474315	0.09	0.01
**Cell Wall proteins**										
gi145324054	45.87	8	33.9	10.7	53.18	arabinogalactan protein 31	38160494	24382141	0.64	0.03
**Nucleic acid binding**										
gi15239697	40	6	41803	4.22	37.45	histone H2A	45987241	19931344	0.43	0.02
gi145333041	22.48	5	41.7	6.55	41.877	glycine-rich RNA-binding protein 2	82983541	11330246	0.14	0.029
**UPREGULATED**										
**Stress response**										
gi334185190	24.35	22	71.1	5.7	202.97	heat shock protein 70-3	72663041	229242810	3.15	0.047
gi15219623	18.32	22	59.7	7.21	162.51	beta-glucosidase 22	21211934	241821111	11.4	0.017
gi145334555	18.65	8	36.9	8.18	66.61	TRAF-like family protein	28420192	72189838	2.54	0.029
gi18421006	20.51	7	39.8	9.62	49.14	TRAF-like family protein	-	45109989	-	unique in *mpk6*
gi15233320	13.6	1	25	5.42	34.18	aquaporin TIP2-1	-	291261	-	unique in *mpk6*
gi238479502	46296	4	50.4	4.93	38.64	jacalin-related lectin 22	-	21204913	-	unique in *mpk6*
**Protein synthesis and folding**										
gi30687350	42.6	9	41.6	4.56	44.45	Elongation factor 1-beta 2	35745455	86882077	2.43	0.007
gi145332383	34.08	15	45.7	6.28	99.12	translational initiation factor 4A-1	6463817	78329209	12.12	0.005
gi15226212	19.83	3	41.6	9.94	31.96	60S ribosomal protein L7-2	9171521	27849796	3.04	0.033
gi30691619	23.13	4	41.6	4.56	26.64	elongation factor 1B beta	-	12710359	-	unique in *mpk6*
gi15231024	41.6	1	59.2	6.3	41.833	T-complex protein 1 subunit alpha	-	6989583	-	unique in *mpk6*
**Protein degradation**										
gi15230005	28.67	2	47.5	5.3	24.69	regulatory particle triple-A ATPase 5A	-	8410216	-	unique in *mpk6*
**Cytoskeletal proteins**										
gi15241179	19.78	11	49.6	5.1	93.14	tubulin alpha-5	27700598	80220285	2.9	0.053

gi15242516	36.34	16	41.7	5.49	127.72	actin 7	23306738	135297066	5.81	0.048
gi30697298	39.52	8	41.6	5.19	47.96	actin depolymerizing factor 3	13497389	38089510	2.82	0.031
gi30683070	28.57	13	47.2	8.9	103.84	tubulin alpha-6 chain	22867734	114613659	5.01	0.022
gi15222075	23.62	12	41.8	5.58	106.86	actin 8	-	114837357	-	unique in *mpk6*
gi15227503	43.07	3	42.1	5.33	46.37	Actin-like ATPase superfamily protein	-	26913061	-	unique in *mpk6*
						**Metabolism**				
gi15222848	52.96	54	36.9	7.18	373.32	glyceraldehyde 3-phosphate dehydrogenase	262612712	595627944	2.27	0.018
gi15229522	35.05	18	53.1	5.74	180.69	adenosylhomocysteinase 2	72720355	115940281	1.56	0.037
gi15236375	41.78	20	51.7	7.23	116.93	serine hydroxymethyltransferase 4	49262480	177373784	3.61	0.007
gi186505986	25.38	4	42.5	6.18	60.44	S-adenosylmethionine synthase 3	7853307	50065528	6.36	0.002
gi334186086	23.62	9	63.8	6.8	61.95	ketol-acid reductoisomerase	9186766	31621802	3.44	0.005
gi26557005	25.44	23	54.9	6.23	175.1	ATPase subunit 1	81707979	154980113	1.9	0.05
gi15231059	24. 53	1	47.5	8.32	18.32	acyl-coenzyme A oxidase 4	-	3900946	-	unique in *mpk6*
gi145332819	46.5	3	51.9	6.7	30.92	pyruvate kinase	-	8527319	-	unique in *mpk6*
						**Lipid metabolism**				
gi15232671	35.09	2	91.8	5.87	56.92	phospholipase D alpha 1	-	5325577	-	unique in *mpk6*
gi15221970	27.39	1	98	5.52	41.69	lipoxygenase 1	-	5459943		
						**Cell division**				
gi15232776	41.7	11	89.3	5.25	70.08	cell division control protein 48-A	-	40140792	-	unique in *mpk6*
						**Aminoacid metabolism**				
gi15233111	46.08	3	39.9	8.54	41.28	cysteine synthase C1	-	11878185	-	unique in *mpk6*
						**Signalling**				
gi18396845	44.16	2	41.7	4.81	41.863	14-3-3 family protein	-	10738972	-	unique in *mpk6*
gi15220941	35.85	1	35.7	7.71	35.27	WD-40 repeat ArcA-like protein (Rack1A)	-	8125602	-	unique in *mpk6*
						**Protein transport**				
gi18391349	16.35	3	41.8	4.15	32.082	vacuolar calcium-binding protein-like protein	-	9683235	-	unique in *mpk6*
						**Unknown function**				
gi145333007	31.35	2	41.6	4.42	24.38	Nascent polypeptide-associated complex subunit alpha-like protein 4	-	9187629	-	unique in *mpk6*
gi15235944	17.98	1	41.8	8.69	41.771	uncharacterized protein	-	32134464	-	unique in *mpk6*

Table S3. Classification of proteins according changes in their abundances in the *mpk4* and *mpk6* mutants, as compared to the Col-0 wild type.

category	*mpk4* mutant	*mpk6* mutant
total number of proteins with significantly changed abundance	65	32
upregulated ≥ 5 fold	9	5
upregulated 1.5- 5 fold	22	11
downregulated 1.5- 5 fold	30	7
downregulated ≥ 5 fold	4	9
detected uniquely in one sample (control/mutant)	25/8	0/19

Table S4. Proteins detected as differentially abundant in both *mpk4* and *mpk6* mutants.

Accession	Protein name	fold change in *mpk4*	fold change in *mpk6*
gi145324054	arabinogalactan protein 31	unique in wt	0.64
gi15233538	profilin 2	0.25	0.21
gi15239697	histone H2A	2.29	0.43
gi18414298	monodehydroascorbate reductase (NADH)	6.1	0.26
gi334185190	heat shock protein 70-3	0.57	3.15
gi145334555	TRAF-like family protein	unique in *mpk4*	2.54
gi15222848	glyceraldehyde 3-phosphate dehydrogenase	2.71	2.27
gi145332383	translational initiation factor 4A-1	2.87	12.11

Table S5. List of differentially regulated proteins in *mpk4* mutant roots containing MAPK-specific phosphorylation site as predicted by GPS 3.0 software.

Position	Code	Kinase	Peptide	Score	Cutoff
**>gi|145324054|ref|NP_001077616.1| arabinogalactan protein 31 (unique in wild type)**					
33	T	CMGC/MAPK	EVNHKTQTPSLAPAP	43.355	35.046
70	S	CMGC/MAPK	HPHPPAKSPVKPPVK	35.092	35.046
81	S	CMGC/MAPK	PPVKAPVSPPAKPPV	39.59	35.046
113	S	CMGC/MAPK	PPVKPPVSPPAKPPV	37.553	35.046
165	S	CMGC/MAPK	APVKPPVSPPTKPPV	41.472	35.046
173	T	CMGC/MAPK	PPTKPPVTPPVYPPK	35.063	35.046
**>gi|30696056|ref|NP_849818.1| elongation factor EF-2-like protein LOS1 (fold change 0.08)**					
764	T	CMGC/MAPK	EEMQRPGTPLYNIKA	35.552	35.046
834	T	CMGC/MAPK	KGLKEAMTPLSEFED	38.873	35.046
**>gi|145325425|ref|NP_001077717.1| proteasome subunit alpha type-5-A (fold change 0.32)**					
56	S	CMGC/MAPK	AVEKRITSPLLEPSS	36.624	35.046
**>gi|30691626|ref|NP_195504.2| heat shock protein 70-1 (fold change 0.32)**					
669	T	CMGC/MAPK	GTSGTEQTPEAEFEE	42.453	35.046
**>gi|334185190|ref|NP_001189847.1| heat shock protein 70-3 (fold change 0.57)**					
403	T	CMGC/MAPK	DLLLLDVTPLSLGLE	48.055	35.046
**>gi|15218090|ref|NP_175610.1| putative mitochondrial-processing peptidase subunit alpha-1 (fold change 2.31)**					
38	T	CMGC/MAPK	VAETSSSTPAYLSWL	37.379	35.046
94	T	CMGC/MAPK	LKIASETTPNPAASI	40.124	35.046
228	S	CMGC/MAPK	GYSGPLASPLYAPES	46.132	35.046
**>gi|15240765|ref|NP_201551.1| voltage dependent anion channel 2 (fold change 2.41)**					
137	S	CMGC/MAPK	DITATLGSPVISFGA	40.513	35.046
**>gi|15221019|ref|NP_175802.1| GDSL esterase/lipase (fold change 5.48)**					
32	S	CMGC/MAPK	ITVAGQNSPVVALFT	36.626	35.046
82	T	CMGC/MAPK	KFSDGLITPDFLAKF	36.566	35.046
371	T	CMGC/MAPK	REFWVPPTPATVHAS	39.119	35.046
**>gi|186513287|ref|NP_001119047.1| argininosuccinate synthase (fold change 3.63)**					
331	S	CMGC/MAPK	ALNGKALSPATLLAE	47.712	35.046
**>gi|15232603|ref|NP_187531.1| 60S acidic ribosomal protein P0-2 (fold change 4.61)**					
158	T	CMGC/MAPK	KGTVEIITPVELIKQ	35.106	35.046
202	S	CMGC/MAPK	YDNGSVFSPEVLDLT	43.1	35.046
**>gi|15232845|ref|NP_186858.1| probable mitochondrial-processing peptidase subunit beta (fold change 13.11)**					
40	S	CMGC/MAPK	DSVPASASPTALSPP	37.688	35.046
45	S	CMGC/MAPK	SASPTALSPPPPHLM	42.61	35.046
**>gi|15226573|ref|NP_179164.1| Ferredoxin--nitrite reductase (unique in wild type)**					
10	S	CMGC/MAPK	SFSLTFTSPLLPSSS	50.472	35.046
**>gi|15233349|ref|NP_195308.1| aconitate hydratase 1 (unique in wild type)**					
304	S	CMGC/MAPK	RATIANMSPEYGATM	36.127	35.046
659	S	CMGC/MAPK	YFKGMTMSPPGPHGV	42.372	35.046
**>gi|18400212|ref|NP_566470.1| dihydrolipoamide acetyltransferase. long form protein (unique in wild type)**					
80	S	CMGC/MAPK	TTSTKLSSPMAGPKL	35.217	35.046
120	S	CMGC/MAPK	EIGMPSLSPTMTEGN	41.945	35.046
**>gi|22331076|ref|NP_566473.2| Subtilase family protein (unique in wild type)**					
21	S	CMGC/MAPK	PLLLCFFSPSSSSSD	37.946	35.046
63	S	CMGC/MAPK	LLRSLPSSPQPATLL	57.857	35.046
85	S	CMGC/MAPK	HGFSARLSPIQTAAL	45.139	35.046
114	T	CMGC/MAPK	REIHTTHTPAFLGFS	41.702	35.046
484	S	CMGC/MAPK	LGTLIGPSPPSPRVA	35.062	35.046
487	S	CMGC/MAPK	LIGPSPPSPRVAAFS	44.914	35.046
713	S	CMGC/MAPK	ANVEIDVSPSKLAFS	37.94	35.046
**>gi|240254562|ref|NP_565741.4| uncharacterized protein (unique in wild type)**					
1299	T	CMGC/MAPK	SSSGNVTTPTQTAST	47.724	35.046
**>gi|30682607|ref|NP_850576.1| mRNA decapping complex VCS (unique in wild type)**					
28	S	CMGC/MAPK	PGISAQPSPVTQQQQ	40.154	35.046
63	S	CMGC/MAPK	TPPLNLQSPRSNHNP	37.132	35.046
625	S	CMGC/MAPK	TLPQLPLSPRLSSKL	59.13	35.046

Table S6. List of differentially regulated proteins in *mpk4* mutant roots containing MAPK-docking site as predicted by The Eukaryotic Linear Motif resource for Functional Sites in Proteins (http://elm.eu.org/).

	**Instances (Matched**	**Sequence)**	**Cell Compartment**	**Pattern**	**Probability**
**arabinogalactan protein 31**	KFNRSLVAV<br>KKLGKSTVVV<br>KLGKSTVVV	180-188<br>285-294<br>286-294	cell wall[1]	[KR]{0,2}[KR].{0,2}[KR].{2,4}[ILVM].[ILVF]	4.324e-03
**elongation factor EF-2**	KRLAKSDPMVV<br>RLAKSDPMVV	509-519<br>510-519	cytoplasm[6]	[KR]{0,2}[KR].{0,2}[KR].{2,4}[ILVM].[ILVF]	4.32E-03
**proteasome subunit alpha type-5-A**	KTKEGVVLAV	41-50	peroxisome, cytoplasm (prediction)	[KR]{0,2}[KR].{0,2}[KR].{2,4}[ILVM].[ILVF]	4.32E-03
**heat shock protein 70-1**	KRSDNIDL<br>KKQLIDL	289-296<br>573-579	cytosol[7]	[KR]{0,2}[KR].{0,2}[KR].{2,4}[ILVM].[ILVF]	4.324e-03
**heat shock protein 70-3**	RARFEELNI<br>RIPKVQQLLV	305-313<br>348-357	nucleus, cytoplasm[2]	[KR]{0,2}[KR].{0,2}[KR].{2,4}[ILVM].[ILVF]	4.324e-03
**putative mitochondrial-processing peptidase subunit alpha-1**	RKMKVEI	196-202	mitochondria	[KR]{0,2}[KR].{0,2}[KR].{2,4}[ILVM].[ILVF]	4.324e-03
**voltage dependent anion channel 2**	KHPRFGLSLAL	264-274	mitochondria plasma membrane[8]	[KR]{0,2}[KR].{0,2}[KR].{2,4}[ILVM].[ILVF]	4.324e-03
**GDSL esterase/lipase**	KFMKIPLAI	88-96	extracellular (prediction)	[KR]{0,2}[KR].{0,2}[KR].{2,4}[ILVM].[ILVF]	4.324e-03
**argininosuccinate synthase**	RGKLKKVVL<br>KKHNVPVPV<br>KKDMYMMSV<br>KLYKGSVSV	93-101<br>255-263<br>293-301<br>393-401	plastid[9]	[KR]{0,2}[KR].{0,2}[KR].{2,4}[ILVM].[ILVF]	4.324e-03
**60S acidic ribosomal protein P0-2**	RKGLRGDSVVL<br>KGLRGDSVVL<br>KINKGTVEI	44-54<br>45-54<br>148-156	ribosome	[KR]{0,2}[KR].{0,2}[KR].{2,4}[ILVM].[ILVF]	4.324e-03
**probable mitochondrial-processing peptidase subunit beta**	RRSQRRLFL<br>RINRERDVIL	11-19<br>210-219	mitochondria	[KR]{0,2}[KR].{0,2}[KR].{2,4}[ILVM].[ILVF]	4.324e-03
**Ferredoxin--nitrite reductase**	KPKRSVLV<br>KIEREPMKL<br>KSSKDDIDVRL<br>RKWNVCV<br>KDGRFGFNLLV<br>KRCEEAIPL<br>RQKTRMMWL<br>KKGVRVTELVPL<br>KGVRVTELVPL	19-26<br>74-82<br>103-113<br>246-252<br>274-284<br>291-299<br>328-336<br>554-565<br>555-565	plastid	[KR]{0,2}[KR].{0,2}[KR].{2,4}[ILVM].[ILVF]	4.324e-03
**aconitate hydratase 1**	RIDKLPYSIRI<br>KRPHDRVPL<br>KKACDLGL	35-45<br>378-386<br>458-465	cytosol, mitochondria[10]	[KR]{0,2}[KR].{0,2}[KR].{2,4}[ILVM].[ILVF]	4.324e-03
**dihydrolipoamide acetyltransferase, long form protein**	RRDHAVAV	20-27	mitochondria[11]	[KR]{0,2}[KR].{0,2}[KR].{2,4}[ILVM].[ILVF]	4.324e-03
**Subtilase family protein**	RRHPSVISV	93-101	extracellular (prediction)	[KR]{0,2}[KR].{0,2}[KR].{2,4}[ILVM].[ILVF]	4.324e-03
**uncharacterized protein**	KSRDIDLSF	1264-1272	nucleus (prediction)	[KR]{0,2}[KR].{0,2}[KR].{2,4}[ILVM].[ILVF]	4.324e-03
**mRNA decapping complex VCS**	RKAQPLVVL<br>KESKRLEVAL<br>KRLEVAL	352-360<br>918-927<br>921-927	cytoplasmic foci[12]	[KR]{0,2}[KR].{0,2}[KR].{2,4}[ILVM].[ILVF]	4.324e-03

Table S7. List of differentially regulated proteins in *mpk6* mutant roots containing MAPK-specific phosphorylation site as predicted by GPS 3.0 software.

Position	Code	Kinase	Peptide	Score	Cutoff
**>gi|15221156|ref|NP_172664.1| pyrophosphate--fructose-6-phosphate 1-phosphotransferase subunit beta 1 (fold change 0.08)**					
16	S	CMGC/MAPK	RDLTAVGSPENAPAK	38.236	35.046
**>gi|145324054|ref|NP_001077616.1| arabinogalactan protein 31 (fold change 0.64)**					
33	T	CMGC/MAPK	EVNHKTQTPSLAPAP	43.355	35.046
70	S	CMGC/MAPK	HPHPPAKSPVKPPVK	35.092	35.046
81	S	CMGC/MAPK	PPVKAPVSPPAKPPV	39.59	35.046
113	S	CMGC/MAPK	PPVKPPVSPPAKPPV	37.553	35.046
165	S	CMGC/MAPK	APVKPPVSPPTKPPV	41.472	35.046
173	T	CMGC/MAPK	PPTKPPVTPPVYPPK	35.063	35.046
**>gi|334185190|ref|NP_001189847.1| heat shock protein 70-3 (fold change 3.15)**					
403	T	CMGC/MAPK	DLLLLDVTPLSLGLE	48.055	35.046
**>gi|15230005|ref|NP_187204.1| regulatory particle triple-A ATPase 5A (unique in *mpk6*)**					
3	T	CMGC/MAPK	*****MATPMVEDTS	48.068	35.046
**>gi|334186086|ref|NP_001190127.1| ketol-acid reductoisomerase (fold change 3.44)**					
16	S	CMGC/MAPK	APSLSCPSPSSSSKT	40.647	35.046
294	S	CMGC/MAPK	GWSVALGSPFTFATT	39.499	35.046
**>gi|15232671|ref|NP_188194.1| phospholipase D alpha 1 (unique in *mpk6*)**					
481	S	CMGC/MAPK	AAAGFPESPEAAAEA	49.609	35.046
**>gi|15232776|ref|NP_187595.1| cell division control protein 48-A (unique in *mpk6*)**					
3	T	CMGC/MAPK	*****MSTPAESSDS	39.309	35.046
**>gi|15233111|ref|NP_191703.1| cysteine synthase C1 (unique in *mpk6*)**					
183	T	CMGC/MAPK	AYDLLDSTPDAFMCQ	35.694	35.046

Table S8. List of differentially regulated proteins in *mpk6* mutant roots containing MAPK-docking site as predicted by The Eukaryotic Linear Motif resource for Functional Sites in Proteins (http://elm.eu.org/).

Protein	Instances (Matched	Sequence)	Cell Compartment	Pattern	Probability
**pyrophosphate--fructose-6-phosphate 1-phosphotransferase subunit beta 1**	KKAMVEL	512-518	plastid, cytoplasm (prediction)	[KR]{0,2}[KR].{0,2}[KR].{2,4}[ILVM].[ILVF]	4.324e-03
**arabinogalactan protein 31**	KFNRSLVAV	180-188	cel wall[1]	[KR]{0,2}[KR].{0,2}[KR].{2,4}[ILVM].[ILVF]	4.324e-03
	KKLGKSTVVV	285-294			
	KLGKSTVVV	286-294			
**heat shock protein 70-3**	RARFEELNI	305-313	nucleus, cytoplasm[2]	[KR]{0,2}[KR].{0,2}[KR].{2,4}[ILVM].[ILVF]	4.324e-03
	RIPKVQQLLV	348-357			
**regulatory particle triple-A ATPase 5A**	RKGKCVVL	102-109	proteasome	[KR]{0,2}[KR].{0,2}[KR].{2,4}[ILVM].[ILVF]	4.324e-03
	KERFEKLGV	194-202			
**ketol-acid reductoisomerase**	KKEKVSL	88-94	plastid (prediction)	[KR]{0,2}[KR].{0,2}[KR].{2,4}[ILVM].[ILVF]	4.324e-03
**phospholipase D alpha 1**	RRPKPGGDVTI	244-254	plasma membrane[3] cytoplasm, nucleus (prediction)	[KR]{0,2}[KR].{0,2}[KR].{2,4}[ILVM].[ILVF]	4.324e-03
	RPKPGGDVTI	245-254			
	KKKASEGVRV	259-268			
	KKASEGVRV	260-268			
	KLRDLSDIII	439-448			
	RRAKDFIYV	511-519			
	RAKDFIYV	512-519			
	KGEKFRVYVVV	561-571			
	KFRVYVVV	564-571			
**cell division control protein 48-A**	RKKSPNRLVV	23-32	nucleus, cytoplasm[4]	[KR]{0,2}[KR].{0,2}[KR].{2,4}[ILVM].[ILVF]	4.324e-03
	KKSPNRLVV	24-32			
	KVVRSNLRVRL	90-100			
	RVRLGDVISV	97-106			
	RPVRKGDLFL	148-157			
	RKGDLFL	151-157			
	KSRAHVIV	339-346			
	RRFGRFDREIDI	361-372			
	RFGRFDREIDI	362-372			
	RFDREIDIGV	365-374			
	KNMKLAEDVDL	389-399			
	REKMDVIDL	427-435			
	RPGRLDQLIYI	639-649			
**cysteine synthase C1**	KRDASLLI	50-57	plastids, cytosol, mitochondria[5]	[KR]{0,2}[KR].{0,2}[KR].{2,4}[ILVM].[ILVF]	4.324e-03
	KSKNPNVKI	238-246			
	KGKLIVTI	331-338			

Table S9. Prediction of potential kinase responsible for phosphorylation of NAI1 (At1g52410) in *Arabidopsis thaliana* as predicted by GPS 3.0 software.

Position	Code	Kinase	Peptide	Score	Cutoff
68	S	CMGC/MAPK	NMNSTSSSPSSSSSS	47.048	14.896
223	S	CMGC/MAPK	SSTCSAASPLSSSSD	41.254	14.896
87	S	CMGC/MAPK	SQVISFGSPDTKTNP	22.791	14.896
67	S	CAMK/CAMKL	NNMNSTSSSPSSSSS	17.138	6.355
98	S	Other/PEK	KTNPVETSLNFSNQV	14.818	6.12
31	T	Other/PEK	DFPICGETNTNPGSE	13.682	6.12
5	S	CK1	***MDDSSFMDLMID	11.75	4.352
229	S	TK	ASPLSSSSDEVSIFK	11.667	8.142
228	S	CMGC/CK2	AASPLSSSSDEVSIF	11.467	7.389
4	S	Other/NEK	****MDDSSFMDLMI	11.321	4.166
58	T	CAMK/CAMKL	RPTKQMKTNNNMNST	11.304	6.355
213	S	CK1/CK1	VYLDDDSSSYSSTCS	11.015	4.847
84	S	Other/WEE	SRTSQVISFGSPDTK	11	3.917
227	S	CMGC/CK2	SAASPLSSSSDEVSI	10.945	7.389
92	T	Other/PEK	FGSPDTKTNPVETSL	10.364	6.12
79	T	Other/Haspin	SSSSGSRTSQVISFG	10	7

Table S10. Examination of the presence of MAPK docking site in the amino acid sequence of NAI1 (At1g52410) as predicted by Eukaryotic Linear Motif (ELM) resource (http://elm.eu.org/index.html).

Elm Name	Instances (Matched Sequence)	Positions	Cell Compartment	Pattern	Probability
DOC_MAPK_1	RKDFGQILI	596-604 [A]	nucleus, cytosol	[KR]{0,2}[KR].{0,2}[KR].{2,4}[ILVM].[ILVF]	4.32E-03

Supplementary References

1. Liu, C. & Mehdy, M. C. A nonclassical arabinogalactan protein gene highly expressed in vascular tissues, AGP31, is transcriptionally repressed by methyl jasmonic acid in Arabidopsis. *Plant Physiol.* **145,** 863–874 (2007).

2. Dufresne, P. J. *et al.* Heat shock 70 protein interaction with Turnip mosaic virus RNA-dependent RNA polymerase within virus-induced membrane vesicles. *Virology* **374,** 217–227 (2008).

3. Gookin, T. E. & Assmann, S. M. Significant reduction of BiFC non-specific assembly facilitates in planta assessment of heterotrimeric G-protein interactors. *Plant J.* **80,** 553–567 (2014).

4. Park, S., Rancour, D. M. & Bednarek, S. Y. In planta analysis of the cell cycle-dependent localization of AtCDC48A and its critical roles in cell division, expansion, and differentiation. *Plant Physiol.* **148,** 246–258 (2008).

5. Heeg, C. *et al.* Analysis of the Arabidopsis O-acetylserine(thiol)lyase gene family demonstrates compartment-specific differences in the regulation of cysteine synthesis. *Plant Cell* **20,** 168–185 (2008).

6. Guo, Y., Xiong, L., Ishitani, M. & Zhu, J.-K. An Arabidopsis mutation in translation elongation factor 2 causes superinduction of CBF/DREB1 transcription factor genes but blocks the induction of their downstream targets under low temperatures. *Proc. Natl. Acad. Sci. U. S. A.* **99,** 7786–7791 (2002).

7. Sung, D. Y., Vierling, E. & Guy, C. L. Comprehensive Expression Profile Analysis of the Arabidopsis Hsp70 Gene Family. *Plant Physiol.* **126,** 789–800 (2001).

8. Robert, N. *et al.* Voltage-dependent-anion-channels (VDACs) in Arabidopsis have a dual localization in the cell but show a distinct role in mitochondria. *Plant Mol. Biol.* **78,** 431–446 (2012).

9. Winter, G., Todd, C. D., Trovato, M., Forlani, G. & Funck, D. Physiological implications of arginine metabolism in plants. *Front. Plant Sci.* **6,** 534 (2015).

10. Hooks, M. A. *et al.* Selective induction and subcellular distribution of ACONITASE 3 reveal the importance of cytosolic citrate metabolism during lipid mobilization in *Arabidopsis*. *Biochem. J.* **463,** 309–317 (2014).

11. Taylor, N. L., Heazlewood, J. L., Day, D. A. & Millar, A. H. Lipoic acid-dependent oxidative catabolism of alpha-keto acids in mitochondria provides evidence for branched-chain amino acid catabolism in Arabidopsis. *Plant Physiol.* **134,** 838–848 (2004).

12. Xu, J., Yang, J.-Y., Niu, Q.-W. & Chua, N.-H. Arabidopsis DCP2, DCP1, and VARICOSE Form a Decapping Complex Required for Postembryonic Development. *Plant Cell* **18,** 3386–3398 (2006).
